# Dietary intake of vitamin A, lung function and incident asthma in childhood

**DOI:** 10.1183/13993003.04407-2020

**Published:** 2021-10-28

**Authors:** Mohammad Talaei, David A. Hughes, Osama Mahmoud, Pauline M. Emmett, Raquel Granell, Stefano Guerra, Seif O. Shaheen

**Affiliations:** 1 Institute of Population Health Sciences, Barts and The London School of Medicine and Dentistry, Queen Mary University of London, London, UK; 2 MRC Integrative Epidemiology Unit, Population Health Sciences, Bristol Medical School, University of Bristol, Bristol, UK; 3 Centre for Academic Child Health, Population Health Sciences, Bristol Medical School, University of Bristol, Bristol, UK; 4 Asthma and Airway Disease Research Center, University of Arizona, Tucson, AZ, USA

## Abstract

**Background:**

Longitudinal epidemiological data are scarce on the relationship between dietary intake of vitamin A and respiratory outcomes in childhood. We investigated whether a higher intake of preformed vitamin A or pro-vitamin β-carotene in mid-childhood is associated with higher lung function and with asthma risk in adolescence.

**Methods:**

In the Avon Longitudinal Study of Parents and Children, dietary intakes of preformed vitamin A and β-carotene equivalents were estimated by food frequency questionnaire at 7 years of age. Post-bronchodilator forced expiratory volume in 1 s (FEV_1_), forced vital capacity (FVC) and forced expiratory flow at 25–75% of FVC (FEF_25–75%_) were measured at 15.5 years and transformed to z-scores. Incident asthma was defined by new cases of doctor-diagnosed asthma at age 11 or 14 years.

**Results:**

In multivariable adjusted models, a higher intake of preformed vitamin A was associated with higher lung function and a lower risk of incident asthma: comparing top *versus* bottom quartiles of intake, regression coefficients for FEV_1_ and FEF_25–75%_ were 0.21 (95% CI 0.05–0.38; p_trend_=0.008) and 0.18 (95% CI 0.03–0.32; p_trend_=0.02), respectively; odds ratios for FEV_1_/FVC below the lower limit of normal and incident asthma were 0.49 (95% CI 0.27–0.90; p_trend_=0.04) and 0.68 (95% CI 0.47–0.99; p_trend_=0.07), respectively. In contrast, there was no evidence for association with β-carotene. We also found some evidence for modification of the associations between preformed vitamin A intake and lung function by *BCMO1*, *NCOR2* and *SCGB1A1* gene polymorphisms.

**Conclusion:**

A higher intake of preformed vitamin A, but not β-carotene, in mid-childhood is associated with higher subsequent lung function and lower risk of fixed airflow limitation and incident asthma.

## Abstract 2

**A higher intake of preformed vitamin A, but not pro-vitamin β-carotene, in mid-childhood was associated with higher subsequent lung function and lower risk of fixed airflow limitation and incident asthma** https://bit.ly/3d7PUca

## Introduction

Vitamin A is a versatile vitamin involved in multiple biological processes including lung development through regulating the expression of several hundred genes [1]. As an essential micronutrient, vitamin A must be obtained from the diet either as preformed vitamin A, comprising mainly retinyl esters in animal foods, or as pro-vitamin A, comprising mainly β-carotene in plant foods [1]. In contrast to preformed vitamin A, β-carotene is converted to retinoids, with different bioconversion abilities partly explained by genetic variability [2].

Animal data suggest that retinoic acid, the ultimate metabolite of vitamin A, plays a crucial role early in life in alveolar development [3], maintenance and regeneration [4], and thus influences elastic recoil [3]; studies also suggest a key role in airway development [5], although evidence for an influence of vitamin A deficiency on airway hyperresponsiveness is conflicting [6, 7]. However, in humans the relationship between dietary vitamin A and respiratory outcomes is not clear, especially in ranges of intake that do not cause severe hypovitaminosis. Follow-up of a trial in a vitamin A-deficient area showed that vitamin A supplementation in early life (peri-pregnancy) improved offspring lung function [8], with no impact on the subsequent risk of asthma [9]. In a birth cohort study in Norway, excess vitamin A intake in pregnancy was associated with a higher risk of childhood asthma [10], while some case–control studies in children have suggested an inverse association between dietary or serum vitamin A and asthma [11]. In adults, an inverse association between serum retinol and subsequent airway obstruction was reported [12], whereas more recently a positive association was found between vitamin A intake and asthma [13].

Maximal attainment of lung function as a young adult through optimal growth is important, as lung function in early adulthood is a powerful predictor of subsequent comorbidities and mortality [14, 15]. Prenatal and early postnatal life are critical time windows for lung development [16], and tracking of lung function from infancy, through childhood, to adulthood has been clearly demonstrated [17–19]. However, reports that alveolarisation continues throughout childhood [20, 21] suggest that catch-up of alveolar growth, at least, is possible beyond infancy. Moreover, recent epidemiological studies have also shown that accelerated growth in forced expiratory volume in 1 s (FEV_1_) occurs in a proportion of children [18, 19]. We know little about environmental influences on catch-up growth [22], and longitudinal epidemiological data on the link between diet in childhood and later respiratory outcomes are scarce [23–26]; in particular, such evidence for dietary intake of vitamin A in childhood is, to the best of our knowledge, absent.

In this study, we have explored the relationships of preformed and pro-vitamin A intake at 7 years of age with lung function and incident asthma up to adolescence. To strengthen causal inference we have also explored whether these associations were modified by polymorphisms linked to bioconversion of β-carotene or metabolism of vitamin A, and by a polymorphism in the gene encoding club cell secretory protein; serum levels of the latter are increased by vitamin A supplementation [27] and positively associated with lung function growth in childhood [28].

## Methods

### Study population

The Avon Longitudinal Study of Parents and Children (ALSPAC) is a population-based birth cohort that recruited predominantly White pregnant women resident in Avon, UK (14 541 pregnancies) with expected dates of delivery from 1 April 1991 to 31 December 1992. The cohort has been followed since birth with annual questionnaires and, since age 7 years, with objective measures in annual research clinics. The study protocol has been described previously [29, 30] and further information can be found at www.alspac.bris.ac.uk, which contains details of all the data that are available (www.bristol.ac.uk/alspac/researchers/our-data). Ethics approval was obtained from the ALSPAC Ethics and Law Committee (IRB 00003312) and the Local National Health Service Research Ethics Committees. Informed consent for the use of data collected *via* questionnaires and clinics was obtained from participants following the recommendations of the ALSPAC Ethics and Law Committee at the time. Consent for biological samples has been collected in accordance with the Human Tissue Act (2004).

### Exposure assessment

We used dietary information collected by food frequency questionnaire (FFQ) at 81 months (∼7 years) of age, which was completed by the child's mother or the main carer. The FFQ included questions about usual consumption of up to 56 food groups and 12 drinks, with five frequency options ranging from “never or rarely” to “more than once a day” and daily consumption of specific types of bread, fat spreads/oils and milk [31]. Standard portion sizes based on typical consumption patterns in Britain [32] were adapted for the age of children and used to estimate the daily intake of each food group. Total energy and nutrient intakes were calculated by multiplying estimated food intake (g·day^−1^) by their estimated nutrient content from UK food composition tables [33, 34] and summing this across all the foods consumed. Accordingly, daily intakes of vitamin A were estimated separately as the intakes of preformed vitamin A and pro-vitamin A carotenes in the form of β-carotene equivalents (sum of β-carotene and half the amounts of α-carotene and α- and β-cryptoxanthins). The major sources of preformed vitamin A were, on average, fat spreads (24.2%), milk (21.6%), cold meats (8.8%), cheese (7.6%), yoghurt (6.1%), liver and liver pate (4.7%), eggs (4.1%), and school meals (4.3%). The major sources of carotene were, on average, carrots (52.1%), other vegetables (10.4%), school meals (8.8%), squash and cordial soft drinks (7.0%), fat spreads (3.2%), fruit (2.3%), and milk (2.2%). We estimated total vitamin A intake by adding intakes of β-carotene equivalents (divided by 12) and preformed vitamin A to give retinol activity equivalents (RAEs) [2].

### Outcome assessment

Lung function was assessed by spirometry (Vitalograph 2120; Vitalograph, Maids Moreton, UK) at 15.5 years, after withholding short-acting bronchodilators for at least 6 h and long-acting bronchodilators and theophyllines for at least 24 h. The best of three reproducible flow–volume curves was used to measure FEV_1_, forced vital capacity (FVC) and forced expiratory flow at 25–75% of FVC (FEF_25–75%_; indicating maximal mid-expiratory flow), before and 15 min after administration of 400 mg salbutamol. These lung function measurements were transformed to z-scores based on the Global Lung Function Initiative (GLI) curves. Accordingly, a standardised measure of an observed lung function measurement was mapped onto the distribution of the population from which the GLI reference values are derived, adjusting for age, height and ethnicity, and separately by sex [35, 36]. The GLI reference values were generated using the GLI R macro (https://github.com/thlytras/rspiro) [37]. The tests adhered to American Thoracic Society criteria for standardisation and reproducibility of flow–volume measurement [38]. We used post-bronchodilator lung function measures as our primary outcomes because, for FEV_1_ and FEF_25–75%_, these are likely to more closely reflect growth and calibre of the airways, rather than airway tone.

Our second primary outcome of interest was incident asthma. At 91 months (∼7.5 years), 128 months (∼11 years) and 166 months (∼14 years) of age, we defined current doctor-diagnosed asthma if mothers responded positively to the question “Has a doctor ever actually said that your study child has asthma?”, and to at least one of the concurrent following questions which asked if the child had had wheezing, wheezing and whistling in the chest, asthma or asthma medication in the last 12 months. Among those children who were not identified as having current doctor-diagnosed asthma at 7.5 years, we defined those with current doctor-diagnosed asthma at 11 or 14 years as cases of incident asthma. The parental reports of a doctor's diagnosis of asthma in ALSPAC agreed well with a general practitioner-recorded diagnosis (sensitivity 88.5% and specificity 95.7%) [39].

### Genotyping and single nucleotide polymorphism selection

We considered single nucleotide polymorphisms (SNPs) that were associated with bioavailability or metabolism of vitamin A in the literature and selected those that could plausibly interact with dietary vitamin A intake and/or have been associated with lung function. We excluded SNPs that were in linkage disequilibrium (r^2^>0.80 using the LDmatrix tool for the British population; https://ldlink.nci.nih.gov) and those with minor allele frequency <0.2 so as not to limit power for stratified analyses. The first SNP of interest (rs3741240) was in the *SCGB1A1* gene, which encodes for the club cell secretory protein CC16 (secretoglobin family 1A member 1). This SNP was shown in a genome-wide association study to have the strongest correlation with serum levels of CC16 [40]. CC16 is an airway epithelial biomarker; serum levels are increased by vitamin A supplementation [27] and are positively associated with lung function growth in childhood [28]. We also included rs12708369 in the *NCOR2* (nuclear receptor corepressor 2) gene, which is found in the retinoic acid signalling pathway and has been associated with FVC in ALSPAC [41]. Among SNPs that were more likely to interact directly with dietary intake and bioavailability [42, 43], we selected five SNPs in the *BCMO1* (β-carotene 15,15′-monooxygenase 1) locus: rs6564851, rs11645428 and rs6420424 (upstream) [44], and rs7501331 and rs12934922 in the coding region [45]. These SNPs have been associated with efficiency of conversion of β-carotene to the intermediate forms of vitamin A and correlated with fasting plasma concentrations of β-carotene, among which rs6564851 had the strongest association [44]. We hypothesised that effects of a higher intake of preformed vitamin A would be greater in poor carotene converters and that effects of a higher intake of carotene would be greater in efficient carotene converters (further details of selected SNPs are given in supplementary table E1). Genotypes were imputed (IMPUTE2) using the Haplotype Reference Consortium genomes reference panel (1.1) and imputation quality was capped (in addition to minor allele frequency) at an imputation information metric score (INFO) >0.95 (further details are given in the supplementary material).

### Statistical analysis

Among 8135 children with plausible data on vitamin A intake at 7 years (excluding children with implausible total energy intake: <15 000 or >140 000 kJ·week^−1^), 2985‒3121 participants had data on post-bronchodilator lung function measures at 15.5 years (depending on the specific measure) and data on incident asthma were complete for 4540 participants (supplementary figure E1). We employed linear regression to examine associations between intakes of preformed vitamin A or carotene (in quartiles) and lung function measures. Logistic regression models were used to test associations with incident asthma and with airflow limitation, defined as an FEV_1_/FVC ratio below the lower limit of normal (LLN), representing the lower 5% of study population z-scores. Linear trend was tested by including median intake of quartiles as a pseudo-continuous variable in the models. We selected known potential confounding factors from the existing literature [46] and by using a directed acyclic graph approach [47] (supplementary figure E2). Details of multivariable models and covariates are explained in the supplementary material.

We carried out stratified analyses, *a priori*, to explore potential modification of dietary associations by maternal and paternal history of atopy (yes/no), maternal smoking when the child was 7 years of age (yes/no), and *SCGB1A1*, *NCOR2* and five *BCMO1* genotypes (supplementary table E1). Potential interactions were assessed by testing cross-product terms of these factors with quartiles (median values) as a continuous factor in regression models. We also carried out several sensitivity analyses, *a priori*, that are explained in the supplementary material in detail, including further adjustment for other potential confounders, restricted cubic spline analysis to examine the dose–response relationship and inverse probability weighting to correct for potential loss-to-follow-up bias [48]. All statistical analyses were performed using Stata version 14.2 (StataCorp, College Station, TX, USA).

## Results

We estimated median (interquartile range) intakes of vitamin A as 429 (332–538) μg·day^−1^ for preformed vitamin A, 1744 (1464–2309) μg·day^–1^ for carotene (β-carotene equivalent) and 590 (470–723) μg·day^–1^ RAE for total vitamin A (comprising a 26.9% contribution by carotene). For comparison with recommended dietary allowances [49], see supplementary figure E3. Table 1 shows that children with higher intakes of preformed vitamin A were more likely to be male, have a smoking mother and have a younger sibling, while less likely to have an older sibling. Mothers of children who had higher intakes of carotene were more educated (supplementary table E2). Children with higher intakes of either preformed vitamin A or carotene had a generally more health-conscious dietary pattern reflected in higher intakes of vitamins C, D and E, and zinc as well as ω-3 from fish, and had higher maternal intakes of vitamin A during pregnancy (table 1 and supplementary table E2).

**TABLE 1:** Participant^#^ characteristics according to quartiles of preformed vitamin A intake at 7 years of age

	**Quartiles of preformed vitamin A intake**				**p-value**
	**Quartile 1**	**Quartile 2**	**Quartile 3**	**Quartile 4**	
**Participants**	1359 (25.2)	1343 (24.9)	1350 (25.1)	1332 (24.7)	
**Preformed vitamin A intake μg·day^–1^**	261±58.4	382±27.6	480±31.3	680±173.6	
**Male**	638 (46.9)	627 (46.7)	663 (49.1)	731 (54.9)	<0.001
**Older siblings**	742 (54.6)	720 (53.6)	686 (50.8)	639 (48.0)	0.002
**Younger siblings**	624 (45.9)	674 (50.2)	717 (53.1)	782 (58.7)	<0.001
**Total energy intake kJ·day^–1^**	6214±1291	7136±1189	7890±1235	9139±1728	<0.001
**BMI kg·m^−2^**	16.2±2.1	16.0±1.9	16.2±2.0	16.2±1.9	0.04
**Health-conscious dietary pattern score**	−0.27±0.89	−0.08±0.88	0.07±0.94	0.33±1.05	<0.001
**Season of dietary information collection**					0.31
Winter	339 (24.9)	354 (26.4)	347 (25.7)	339 (25.5)	
Spring	424 (31.2)	393 (29.3)	398 (29.5)	370 (27.8)	
Summer	366 (26.9)	373 (27.8)	381 (28.2)	395 (29.7)	
Autumn	207 (15.2)	214 (15.9)	214 (15.9)	213 (16.0)	
Missing	23 (1.7)	9 (0.7)	10 (0.7)	15 (1.1)	
**History of food allergy**	263 (19.4)	219 (16.3)	221 (16.4)	234 (17.6)	0.12
**Any supplement use**	450 (33.1)	440 (32.8)	453 (33.6)	461 (34.6)	0.76
**Protein intake g·day^−1^**	52.8±12.2	61.1±11.5	66.7±11.6	77.2±15.8	<0.001
**Vitamin C intake mg·day^–1^**	68.2±31.4	72.9±32.5	78.2±33.0	85.3±35.8	<0.001
**Vitamin D intake mg·day^–1^**	2.14±0.7	2.69±0.8	3.01±0.9	3.45±1.1	<0.001
**Vitamin E intake mg·day^–1^**	7.49±2.6	9.23±2.9	10.32±3.3	11.83±4.0	<0.001
**Zinc intake mg·day^–1^**	5.19±1.3	5.99±1.3	6.57±1.3	7.66±1.8	<0.001
**VLC *n*-3 PUFA intake from fish mg·day^–1^**	65.9±73.3	77.9±84.6	83.8±91.0	98.7±99.2	<0.001
**Parental factors**					
** **Maternal age at pregnancy years	29.5±4.4	29.4±4.4	29.5±4.4	29.1±4.5	0.02
** **Maternal education					0.21
Secondary or vocational	281 (20.7)	267 (19.9)	237 (17.6)	236 (17.7)	
O-level	464 (34.1)	450 (33.5)	476 (35.3)	451 (33.9)	
A-level or degree	593 (43.6)	608 (45.3)	624 (46.2)	619 (46.5)	
Missing	21 (1.5)	18 (1.3)	13 (1.0)	26 (2.0)	
** **Housing tenure during pregnancy					
Mortgaged/owned	1156 (85.1)	1136 (84.6)	1151 (85.3)	1071 (80.4)	
Council rented	74 (5.4)	71 (5.3)	74 (5.5)	107 (8.0)	
Non-council rented	74 (5.4)	71 (5.3)	68 (5.0)	91 (6.8)	
Missing	55 (4.0)	65 (4.8)	57 (4.2)	63 (4.7)	
** **Financial difficulty during pregnancy					0.24
No	1156 (85.3)	1136 (85.4)	1153 (85.9)	1105 (83.3)	
Yes	200 (14.8)	195 (14.7)	189 (14.1)	222 (16.7)	
** **Maternal ethnicity					0.02
White	1307 (96.2)	1297 (96.6)	1324 (98.1)	1292 (97.0)	
Non-White	25 (1.8)	27 (2.0)	11 (0.8)	14 (1.1)	
Missing	27 (2.0)	19 (1.4)	15 (1.1)	26 (2.0)	
** **Maternal history of atopy					0.12
No	713 (52.5)	712 (53.0)	700 (51.9)	652 (48.9)	
Yes	594 (43.7)	579 (43.1)	613 (45.4)	622 (46.7)	
Missing	52 (3.8)	52 (3.9)	37 (2.7)	58 (4.4)	
** **Paternal history of atopy					0.02
No	584 (43.0)	601 (44.8)	553 (41.0)	582 (43.7)	
Yes	414 (30.5)	385 (28.7)	470 (34.8)	388 (29.1)	
Missing	361 (26.6)	357 (26.6)	327 (24.2)	362 (27.2)	
** **Maternal smoking					0.01
No	1085 (79.8)	1102 (82.1)	1100 (81.5)	1020 (76.6)	
Yes	219 (16.1)	194 (14.4)	201 (14.9)	257 (19.3)	
Missing	55 (4.0)	47 (3.5)	49 (3.6)	55 (4.1)	
** **Preformed vitamin A intake at 32 weeks of gestation μg·day^–1^	302±249	335±276	357±319	414±379	<0.001

Data are presented as n (%) or mean±sd, unless otherwise specified. BMI: body mass index; VLC *n*-3 PUFA: very-long-chain ω-3 polyunsaturated fatty acid. ^#^: children included in incident asthma or lung function analysis (n=5384).

### Lung function

Higher intake of preformed vitamin A was associated with a higher FEV_1_ and FEF_25–75%_. There was also weak evidence of association with FVC, but not with FEV_1_/FVC, analysed as a continuous variable (table 2). However, when we analysed the dichotomous outcome of airflow limitation (defined as FEV_1_/FVC <LLN), higher intake was inversely associated (OR comparing top *versus* bottom quartile in model 2: 0.49, 95% CI 0.27–0.90; p_trend_=0.04). We did not find any evidence of association between carotene intake and lung function measures overall (table 2).

**TABLE 2:** Linear regression coefficients (95% CI) for post-bronchodilator lung function measures (z-scores) according to quartiles of intakes of preformed vitamin A and β-carotene equivalent, adjusted for potential confounders

	**Quartiles of vitamin A intake**				**p_trend_-value** ^#^	**Per sd**
	**Quartile 1**	**Quartile 2**	**Quartile 3**	**Quartile 4**		
**Preformed vitamin A median (IQR) mg·day^–1^**	276 (224–305)	382 (359–407)	477 (452–506)	637 (581–721)		
**FEV_1_**						
Model 1	0.00	−0.01 (−0.15–0.13)	−0.01 (−0.16–0.13)	0.20 (0.03–0.36)	0.02	0.08 (0.01–0.14)
Model 2	0.00	−0.02 (−0.16–0.12)	−0.01 (−0.15–0.14)	0.21 (0.05–0.38)	0.008	0.09 (0.02–0.15)
**FVC**						
Model 1	0.00	−0.01 (−0.14–0.13)	−0.03 (−0.17–0.11)	0.14 (−0.02–0.30)	0.09	0.05 (−0.02–0.11)
Model 2	0.00	−0.01 (−0.14–0.13)	−0.02 (−0.16–0.12)	0.15 (−0.01–0.31)	0.06	0.05 (−0.01–0.11)
**FEV_1_/FVC**						
Model 1	0.00	0.02 (−0.10–0.14)	0.06 (−0.06–0.19)	0.07 (−0.07–0.21)	0.30	0.04 (−0.02–0.09)
Model 2	0.00	0.01 (−0.11–0.13)	0.05 (−0.07–0.18)	0.08 (−0.06–0.22)	0.21	0.05 (−0.01–0.10)
**FEF_25–75%_**						
Model 1	0.00	0.04 (−0.07–0.16)	0.05 (−0.08–0.17)	0.16 (0.02–0.30)	0.03	0.07 (0.02–0.13)
Model 2	0.00	0.04 (−0.08–0.16)	0.05 (−0.08–0.17)	0.18 (0.03–0.32)	0.02	0.08 (0.03–0.14)
**β-carotene equivalent** **median (IQR) mg·day^–1^**	956 (646–1328)	1607 (1538–1671)	1945 (1827–2105)	3268 (2670–3616)		
**FEV_1_**						
Model 1	0.00	0.07 (−0.06–0.21)	0.09 (−0.06–0.23)	0.00 (−0.14–0.15)	0.71	−0.00 (−0.06–0.05)
Model 2	0.00	0.07 (−0.07–0.20)	0.10 (−0.05–0.24)	0.01 (−0.14–0.15)	0.77	−0.00 (−0.05–0.05)
**FVC**						
Model 1	0.00	0.03 (−0.10–0.16)	0.05 (−0.09–0.18)	−0.02 (−0.16–0.12)	0.61	−0.01 (−0.06–0.04)
Model 2	0.00	0.03 (−0.10–0.16)	0.06 (−0.08–0.19)	−0.01 (−0.16–0.13)	0.68	−0.01 (−0.06–0.04)
**FEV_1_/FVC**						
Model 1	0.00	0.07 (−0.04–0.19)	0.03 (−0.09–0.15)	−0.02 (−0.15–0.10)	0.42	−0.01 (−0.05–0.03)
Model 2	0.00	0.05 (−0.06–0.17)	0.02 (−0.10–0.14)	−0.04 (−0.17–0.09)	0.33	−0.01 (−0.06–0.03)
**FEF_25–75%_**						
Model 1	0.00	0.09 (−0.03–0.21)	0.09 (−0.04–0.21)	0.03 (−0.09–0.16)	0.95	0.00 (−0.04–0.05)
Model 2	0.00	0.08 (−0.04–0.19)	0.08 (−0.04–0.20)	0.02 (−0.11–0.15)	0.92	0.00 (−0.04–0.05)

IQR: interquartile range; FEV_1_: forced expiratory volume in 1 s; FVC: forced vital capacity; FEF_25–75%_: forced expiratory flow at 25–75% of FVC. Multivariable model 1: sex and total energy intake. Multivariable model 2: further adjusted for maternal education, housing tenure at birth, financial difficulty during pregnancy, maternal ethnicity, maternal history of atopic disease, paternal history of atopic disease, maternal smoking, older sibling, younger sibling, supplement use and season when the food frequency questionnaire was completed. ^#^: linear trend was tested by treating the median values of quartiles as a continuous variable.

There was no evidence of interaction with maternal atopy, paternal atopy or maternal smoking (data not shown). We also tested relationships with pre-bronchodilator lung function measures, and found the same pattern of associations between preformed vitamin A intake and FEV_1_ and FEF_25–75%_, although slightly weaker (supplementary table E3).

In stratified analysis by *SCGB1A1* genotype (rs3741240), there were positive associations between preformed vitamin A intake and FEF_25–75%_ and FEV_1_/FVC only in homozygous carriers of the G allele (p_interaction_≤0.02), and the suggestion of a similar pattern with FEV_1_ (p_interaction_=0.07), while no evidence of effect modification was observed for β-carotene intake (table 3). When we stratified by *NCOR2* genotype (rs12708369), preformed vitamin A intake was positively associated with FEV_1_ and FVC in carriers of the C allele, but negatively associated in those homozygous for the T allele (p_interaction_≤0.02 and ≤0.01, respectively) (table 4). A similar pattern of associations was observed between carotene intake and FEF_25–75%_ in those homozygous for the C and T allele, respectively (p_interaction_=0.009).

**TABLE 3:** Linear regression coefficients (95% CI) for post-bronchodilator lung function measures (z-scores) according to quartiles of intakes of preformed vitamin A and β**-**carotene equivalent, stratified by *SCGB1A1* (club cell secretory protein CC16)^#^ genotype (rs3741240)

	**Quartiles of vitamin A intake**				**p_trend_-value** ^¶^	**p_interaction_-value**
	**Quartile 1**	**Quartile 2**	**Quartile 3**	**Quartile 4**		
**Preformed vitamin A**						
**FEV_1_**						
Genotype GG	0.00	−0.12 (−0.37–0.12)	0.01 (−0.24–0.27)	0.27 (−0.01–0.56)	0.02	
Genotype GA	0.00	−0.02 (−0.25–0.20)	−0.05 (−0.28–0.19)	0.13 (−0.14–0.39)	0.33	0.35
Genotype AA	0.00	0.10 (−0.30–0.50)	0.02 (−0.39–0.43)	−0.09 (−0.57–0.38)	0.65	0.07
**FVC**						
Genotype GG	0.00	−0.12 (−0.35–0.12)	−0.04 (−0.28–0.20)	0.11 (−0.16–0.39)	0.24	
Genotype GA	0.00	0.02 (−0.19–0.23)	−0.02 (−0.24–0.20)	0.11 (−0.15–0.36)	0.43	0.88
Genotype AA	0.00	0.12 (−0.27–0.52)	0.11 (−0.29–0.51)	0.05 (−0.42–0.52)	0.85	0.42
**FEV_1_/FVC**						
Genotype GG	0.00	0.07 (−0.13–0.27)	0.19 (−0.02–0.40)	0.32 (0.08–0.55)	0.004	
Genotype GA	0.00	−0.05 (−0.25–0.15)	0.02 (−0.18–0.23)	0.00 (−0.24–0.24)	0.86	0.15
Genotype AA	0.00	−0.03 (−0.36–0.30)	−0.24 (−0.58–0.09)	−0.29 (−0.68–0.10)	0.09	0.02
**FEF_25–75%_**						
Genotype GG	0.00	−0.02 (−0.23–0.19)	0.08 (−0.13–0.30)	0.31 (0.07–0.55)	0.004	
Genotype GA	0.00	−0.01 (−0.20–0.18)	−0.03 (−0.23–0.17)	0.10 (−0.13–0.33)	0.40	0.35
Genotype AA	0.00	0.13 (−0.21–0.48)	0.08 (−0.27–0.43)	−0.27 (−0.68–0.13)	0.18	0.01
**β-** **carotene equivalent**						
**FEV_1_**						
Genotype GG	0.00	0.05 (−0.18–0.29)	0.12 (−0.13–0.36)	0.10 (−0.15–0.35)	0.48	
Genotype GA	0.00	0.04 (−0.17–0.26)	0.21 (−0.02–0.43)	−0.04 (−0.27–0.19)	0.52	0.36
Genotype AA	0.00	−0.04 (−0.45–0.37)	−0.06 (−0.50–0.39)	0.15 (−0.31–0.61)	0.36	0.88
**FVC**						
Genotype GG	0.00	−0.10 (−0.32–0.13)	0.06 (−0.18–0.29)	−0.02 (−0.26–0.22)	0.99	
Genotype GA	0.00	0.07 (−0.13–0.28)	0.14 (−0.07–0.36)	0.05 (−0.18–0.27)	0.87	0.93
Genotype AA	0.00	−0.03 (−0.43–0.37)	−0.07 (−0.50–0.36)	0.05 (−0.40–0.50)	0.71	0.94
**FEV_1_/FVC**						
Genotype GG	0.00	0.18 (−0.02–0.37)	0.04 (−0.16–0.24)	0.07 (−0.14–0.27)	0.80	
Genotype GA	0.00	0.02 (−0.18–0.21)	0.08 (−0.12–0.28)	−0.16 (−0.37–0.05)	0.06	0.21
Genotype AA	0.00	−0.06 (−0.39–0.28)	−0.00 (−0.37–0.36)	0.09 (−0.29–0.47)	0.46	0.55
**FEF_25–75%_**						
Genotype GG	0.00	0.16 (−0.04–0.36)	0.10 (−0.11–0.30)	0.15 (−0.06–0.37)	0.25	
Genotype GA	0.00	0.03 (−0.15–0.22)	0.17 (−0.03–0.36)	−0.01 (−0.21–0.19)	0.69	0.43
Genotype AA	0.00	−0.09 (−0.44–0.27)	−0.03 (−0.40–0.35)	0.03 (−0.37–0.43)	0.69	0.66

FEV_1_: forced expiratory volume in 1 s; FVC: forced vital capacity; FEF_25–75%_: forced expiratory flow at 25–75% of FVC. Multivariable model: sex, total energy intake, maternal education, housing tenure at birth, financial difficulty during pregnancy, maternal ethnicity, maternal history of atopic disease, paternal history of atopic disease, maternal smoking, older sibling, younger sibling, supplement use and season when the food frequency questionnaire was completed. ^#^: in GG, GA and AA groups, sample sizes were 1074, 1133 and 348 for FEV_1_, and 1126, 1183 and 360 for both FVC and FEF_25–75%_, respectively; ^¶^: linear trend was tested by treating the median values of quartiles as a continuous variable.

**TABLE 4:** Linear regression coefficients (95% CI) for post-bronchodilator lung function measures (z-scores) according to quartiles of intakes of preformed vitamin A and β**-**carotene equivalent, stratified by *NCOR2* (nuclear receptor corepressor 2)^#^ genotype (rs12708369)

	**Quartiles of vitamin A intake**				**p_trend_-value** ^¶^	**p_interaction_-value**
	**Quartile 1**	**Quartile 2**	**Quartile 3**	**Quartile 4**		
**Preformed vitamin A**						
**FEV_1_**						
Genotype TT	0.00	−0.41 (−0.81– −0.02)	−0.27 (−0.65–0.10)	−0.55 (−1.03– −0.07)	0.046	
Genotype CT	0.00	−0.03 (−0.26–0.19)	0.08 (−0.15–0.31)	0.30 (0.04–0.56)	0.01	0.01
Genotype CC	0.00	0.01 (−0.23–0.26)	0.01 (−0.25–0.26)	0.29 (−0.01–0.58)	0.047	0.02
**FVC**						
Genotype TT	0.00	−0.23 (−0.61–0.15)	−0.29 (−0.66–0.07)	−0.63 (−1.09– −0.17)	0.008	
Genotype CT	0.00	−0.05 (−0.26–0.16)	0.07 (−0.15–0.29)	0.25 (0.01–0.50)	0.02	0.003
Genotype CC	0.00	0.06 (−0.18–0.29)	0.04 (−0.20–0.28)	0.23 (−0.05–0.51)	0.11	0.01
**FEV_1_/FVC**						
Genotype TT	0.00	−0.24 (−0.60–0.13)	0.08 (−0.26–0.43)	0.05 (−0.39–0.49)	0.54	
Genotype CT	0.00	0.10 (−0.08–0.29)	0.09 (−0.11–0.28)	0.15 (−0.07–0.37)	0.23	0.73
Genotype CC	0.00	−0.04 (−0.26–0.17)	−0.00 (−0.23–0.22)	0.01 (−0.24–0.27)	0.84	0.66
**FEF_25–75%_**						
Genotype TT	0.00	−0.34 (−0.68–0.00)	−0.02 (−0.35–0.30)	−0.14 (−0.55–0.27)	0.84	
Genotype CT	0.00	0.09 (−0.09–0.28)	0.11 (−0.09–0.30)	0.25 (0.03–0.47)	0.03	0.37
Genotype CC	0.00	−0.05 (−0.27–0.17)	−0.05 (−0.28–0.18)	0.10 (−0.16–0.36)	0.39	0.32
**β-** **carotene equivalent**						
**FEV_1_**						
Genotype TT	0.00	−0.44 (−0.82– −0.05)	−0.11 (−0.52–0.29)	−0.26 (−0.66–0.15)	0.40	
Genotype CT	0.00	0.09 (−0.12–0.31)	0.08 (−0.14–0.30)	−0.07 (−0.31–0.16)	0.33	0.88
Genotype CC	0.00	0.15 (−0.09–0.38)	0.27 (0.01–0.52)	0.27 (0.02–0.53)	0.06	0.23
**FVC**						
Genotype TT	0.00	−0.54 (−0.91– −0.17)	−0.16 (−0.55–0.23)	−0.15 (−0.54–0.24)	0.89	
Genotype CT	0.00	0.09 (−0.11–0.30)	0.10 (−0.11–0.31)	−0.04 (−0.27–0.18)	0.46	0.61
Genotype CC	0.00	0.03 (−0.19–0.26)	0.12 (−0.12–0.37)	0.13 (−0.12–0.37)	0.33	0.81
**FEV_1_/FVC**						
Genotype TT	0.00	0.18 (−0.17–0.53)	0.03 (−0.35–0.40)	−0.31 (−0.68–0.06)	0.03	
Genotype CT	0.00	0.05 (−0.13–0.23)	−0.07 (−0.25–0.12)	−0.08 (−0.28–0.11)	0.30	0.35
Genotype CC	0.00	0.14 (−0.06–0.35)	0.20 (−0.02–0.42)	0.15 (−0.08–0.37)	0.35	0.07
**FEF_25–75%_**						
Genotype TT	0.00	−0.04 (−0.37–0.29)	−0.09 (−0.44–0.26)	−0.36 (−0.71– −0.01)	0.03	
Genotype CT	0.00	0.00 (−0.18–0.19)	0.02 (−0.16–0.21)	−0.03 (−0.23–0.17)	0.73	0.25
Genotype CC	0.00	0.22 (0.01–0.44)	0.28 (0.05–0.51)	0.30 (0.07–0.53)	0.03	0.009

FEV_1_: forced expiratory volume in 1 s; FVC: forced vital capacity; FEF_25–75%_: forced expiratory flow at 25–75% of FVC. Multivariable model: sex, total energy intake, maternal education, housing tenure at birth, financial difficulty during pregnancy, maternal ethnicity, maternal history of atopic disease, paternal history of atopic disease, maternal smoking, older sibling, younger sibling, supplement use and season when the food frequency questionnaire was completed. ^#^: in TT, CT and TT groups, sample sizes were 380, 1227 and 948 for FEV_1_, and 397, 1287 and 985 for both FVC and FEF_25–75%_, respectively; ^¶^: linear trend was tested by treating the median values of quartiles as a continuous variable.

We also found evidence of effect modification by two of the SNPs in the coding region of *BCMO1*: in carriers of the T allele of rs7501331 (low converters of β-carotene), but not in those homozygous for the C allele, higher intake of preformed vitamin A was associated with higher FEV_1_ and FVC (p_interaction_=0.03 and 0.01, respectively), whereas in carriers of the A allele of rs12934922 (high converters of β-carotene) higher intake of β-carotene was associated with higher FEV_1_ (p_interaction_=0.01) (supplementary table E4). We did not find any other convincing evidence of interaction with the other four *BCMO1* SNPs (data not shown).

### Asthma

We identified 390 (8.6%) cases of incident asthma at 11 or 14 years. There was weak evidence of an inverse association between preformed vitamin A intake and incident asthma (table 5). When stratified by paternal history of atopy, we found evidence of lower risk of asthma with higher intakes of preformed vitamin A in children without a paternal history of atopy (OR comparing top *versus* bottom quartile: 0.52, 95% CI 0.28–0.97; p_trend_=0.03), but not in those with it (OR 1.36, 95% CI 0.73–2.55; p_trend_=0.19) (p_interaction_=0.02). We did not find any evidence of association between carotene intake and incident asthma overall (table 5) nor any other evidence of interaction with non-genetic factors (data not shown).

**TABLE 5:** Odds ratios (95% CI) for incident asthma at 11 or 14** **years according to quartiles of intakes of preformed vitamin A and β-carotene equivalent, adjusted for potential confounders

	**Quartiles of vitamin A intake**				**p_trend_-value^#^**	**Per sd**
	**Quartile 1**	**Quartile 2**	**Quartile 3**	**Quartile 4**		
**Preformed vitamin A**						
Cases/non-cases	108/1026	90/1063	99/1037	93/1024		
Model 1	1.00	0.76 (0.56–1.02)	0.81 (0.59–1.11)	0.70 (0.49–1.01)	0.10	0.83 (0.71–0.97)
Model 2	1.00	0.77 (0.57–1.04)	0.81 (0.59–1.10)	0.68 (0.47–0.99)	0.07	0.82 (0.70–0.96)
**β-** **carotene equivalent**						
Cases/non-cases	95/1023	76/1044	111/1061	108/1022		
Model 1	1.00	0.78 (0.57–1.07)	1.12 (0.83–1.51)	1.12 (0.82–1.54)	0.26	1.06 (0.95–1.18)
Model 2	1.00	0.80 (0.58–1.10)	1.15 (0.84–1.56)	1.16 (0.85–1.60)	0.20	1.07 (0.96–1.20)

Data for case/non-cases are presented as n/n. Multivariable model 1: sex and total energy intake. Multivariable model 2: further adjusted for maternal education, housing tenure at birth, financial difficulty during pregnancy, maternal ethnicity, maternal history of atopic disease, paternal history of atopic disease, maternal smoking, older sibling, younger sibling, supplement use and season when the food frequency questionnaire was completed. ^#^: linear trend was tested by treating the median values of quartiles as a continuous variable.

There was an inverse association between preformed vitamin A intake and incident asthma in individuals with an upstream *BCMO1* SNP genotype associated with poor conversion of carotene (rs6564851_GG), but also in individuals with a coding region *BCMO1* SNP genotype associated with high conversion (rs7501331_CC). However, there was no evidence of statistically significant interaction by genotype (table 6). In contrast, there was a positive association between carotene intake and incident asthma in individuals with an upstream *BCMO1* SNP genotype associated with high conversion (rs6564851_TT), but also in individuals with another coding region *BCMO1* SNP genotype associated with low conversion (rs12934922_TT) (table 6). We found a similar pattern of associations when stratified by other *BCMO1* SNPs (supplementary table E5).

**TABLE 6:** Adjusted odds ratios (aORs) (95% CI) for incident asthma at 11 or 14** **years according to quartiles of intakes of preformed vitamin A and β-carotene equivalent, stratified by *BCMO1* (β-carotene 15,15′-monooxygenase 1) genotypes

	**Quartiles of vitamin A intake**				**p_trend_-value** ^#^	**p_interaction_-value**
	**Quartile 1**	**Quartile 2**	**Quartile 3**	**Quartile 4**		
**Preformed vitamin A**						
**Upstream *BCMO1*: rs6564851**						
TT^¶^: cases/non-cases	16/189	14/205	17/191	17/188		
aOR (95% CI)	1.00	0.73 (0.32–1.66)	1.01 (0.44–2.30)	0.89 (0.35–2.28)	0.99	
TG: cases/non-cases	41/389	36/421	40/397	45/378		
aOR (95% CI)	1.00	0.87 (0.54–1.43)	0.96 (0.58–1.58)	1.12 (0.63–1.98)	0.61	0.92
GG: cases/non-cases	26/221	16/240	25/249	20/250		
aOR (95% CI)	1.00	0.43 (0.21–0.85)	0.59 (0.31–1.13)	0.34 (0.15–0.77)	0.03	0.48
***BCMO1* coding region: rs7501331**						
CC^¶^: cases/non-cases	53/463	34/512	53/466	45/515		
aOR (95% CI)	1.00	0.49 (0.31–0.78)	0.80 (0.51–1.25)	0.47 (0.28–0.81)	0.04	
CT: cases/non-cases	25/285	26/303	25/324	29/255		
aOR (95% CI)	1.00	1.08 (0.59–2.00)	0.94 (0.49–1.81)	1.31 (0.63–2.73)	0.51	0.23
TT: cases/non-cases	5/51	6/51	<5/47	8/46		
aOR (95% CI)	1.00	0.86 (0.16–4.73)	0.93 (0.16–5.27)	6.85 (0.91–51.7)	0.06	0.41
**β-** **carotene equivalent**						
**Upstream *BCMO1*: rs6564851**						
TT^¶^: cases/non-cases	7/192	13/215	15/190	29/176		
aOR (95% CI)	1.00	2.00 (0.75–5.31)	2.70 (1.01–7.19)	5.20 (2.04–13.27)	<0.001	
TG: cases/non-cases	45/375	34/404	37/403	46/403		
aOR (95% CI)	1.00	0.72 (0.45–1.17)	0.77 (0.47–1.27)	0.93 (0.57–1.51)	0.93	0.001
GG: cases/non-cases	22/228	16/232	33/252	16/248		
aOR (95% CI)	1.00	0.61 (0.31–1.23)	1.08 (0.58–1.99)	0.51 (0.24–1.08)	0.10	<0.001
***BCMO1* coding region: rs12934922**						
AA^¶^: cases/non-cases	25/244	28/257	24/279	24/272		
aOR (95% CI)	1.00	1.14 (0.63–2.05)	0.83 (0.44–1.59)	0.91 (0.47–1.76)	0.71	
AT: cases/non-cases	36/394	27/422	40/388	38/404		
aOR (95% CI)	1.00	0.68 (0.40–1.15)	1.03 (0.62–1.72)	0.90 (0.53–1.53)	0.89	0.43
TT: cases/non-cases	13/157	8/172	21/178	29/151		
aOR (95% CI)	1.00	0.62 (0.24–1.58)	1.67 (0.76–3.67)	2.52 (1.15–5.52)	0.003	0.005

Data for case/non-cases are presented as n/n. Adjusted odds ratio (multivariable model) for sex, total energy intake, maternal education, housing tenure at birth, financial difficulty during pregnancy, maternal ethnicity, maternal history of atopic disease, paternal history of atopic disease, maternal smoking, older sibling, younger sibling, supplement use and season when the food frequency questionnaire was completed. ^#^: linear trend was tested by treating the median values of quartiles as a continuous variable; ^¶^: homozygous alleles linked to a more efficient conversion of carotene pro-vitamin A.

### Sensitivity analyses

We did not find evidence of any nonlinear associations, except between preformed vitamin A and FEV_1_ (p_nonlinearity_=0.04) using the restricted cubic spline analysis. The associations between intake of preformed vitamin A and FEV_1_, FEF_25–75%_ and incident asthma did not materially change after further adjustment for dietary patterns (“health-conscious”, “junk” and “traditional”, separately), any history of food allergy, breastfeeding, urban/rural locality, physical activity, body mass index (imputed for 8.7–12.1% missing data), atopy measured by skin prick test and maternal intake of vitamin A in pregnancy, as well as other dietary factors including intakes of vitamins C, D and E, zinc, protein, and *n*-3 fatty acids from fish (supplementary tables E6 and E8). The null associations for carotene intake also remained the same after these further adjustments (supplementary tables E7 and E8). When we tested energy-adjusted intakes using the residual method, preformed vitamin A was almost similarly associated with FEV_1_ and FEF_25–75%_ (multivariable adjusted regression coefficients per sd: 0.07, 95% CI 0.02–0.12 and 0.07, 95% CI 0.02–0.11, respectively) and incident asthma (multivariable adjusted OR per sd: 0.85, 95% CI 0.75–0.97), the associations with FVC and FEV_1_/FVC did not change either, and no association was observed for carotene intake (supplementary table E9).

When we excluded those with asthma at 7 or 14 years, the associations between dietary vitamin A and lung function outcomes did not materially change. For lung function outcomes and incident asthma, findings did not materially change after exclusion of children of non-White mothers (2.5–3%), those with a history of food allergy (15.3–18.4%), those with extreme energy intakes or those with a history of consuming vitamin A-containing supplements (11.6–12.8%). Among eligible children with data on vitamin A intake at 7 years of age, 25.5% and 55.9% did not have data on incident asthma or lung function at 15.5 years, respectively. However, findings were similar when we applied inverse probability weighting to correct for selection bias due to loss to follow-ups (data not shown).

## Discussion

In ALSPAC children overall we found that higher intake of preformed vitamin A, but not β-carotene, in mid-childhood was associated with a higher subsequent FEV_1_ and FEF_25–75%_. There was also weak evidence for an inverse association between intake of preformed vitamin A and incident asthma. To the best of our knowledge, these are novel findings, which were robust to various sensitivity analyses.

The difference in FEV_1_ between the top and bottom quartiles of preformed vitamin A intake was clinically important and comparable to the mean difference in z-scores of pre-bronchodilator FEV_1_ according to asthma status (0.24, 95% CI 0.11–0.37). Associations between preformed vitamin A intake and lung function were stronger for FEV_1_ and FEF_25–75%_ than for FVC, suggesting a stronger influence on airway than alveolar development. Furthermore, the stronger associations with post- than pre-bronchodilator measures suggest that higher intake may promote growth and calibre, rather than tone, of large and small airways. The weak inverse association with asthma may therefore also reflect a beneficial effect on airway growth. Moreover, the strong inverse association with fixed airflow limitation (FEV_1_/FVC <LLN) may have implications for the development of later chronic obstructive pulmonary disease.

Vitamin A is the most multifunctional vitamin in the human body and the only one with a storage system buffering against dietary insufficiency, which underlines its evolutionary importance [1]. Overt vitamin A deficiency is mostly a problem in poorly nourished populations, due to the lower consumption of animal foods. While in the developed world it is estimated that >20% of the population may not meet the recommended intake due to modern societal habits [1], the estimated level of total vitamin A intake in this study was higher than the recommended dietary allowance for children 4–8 years of age (400 µg·day^–1^ RAE) [49]. An intestinal negative feedback loop restricts β-carotene absorption and cleavage in response to vitamin A status [42]. Therefore, the lack of association between β-carotene and lung function outcomes might be explained by its limited contribution to vitamin A status in this population, which is in line with other Western societies (<30%) [2].

Previous findings on the link between serum concentration of retinol or β-carotene and lung function were in adults and were conflicting [12, 50]. However, the serum concentration of vitamin A biomarkers reflects a combined effect of dietary intake, bioavailability and metabolism. Vitamin A is mainly stored in the liver which tightly regulates the circulatory level of retinol; the latter does not decline until the liver is almost depleted [1]. Nevertheless, our findings suggest that, even in a Western population of children without overt vitamin A deficiency, higher intakes of vitamin A may beneficially influence lung growth and hence optimal lung function attainment.

### Mechanism

We found evidence for effect modification of the association between preformed vitamin A intake and lung function by a *SCGB1A1* polymorphism that has been shown to regulate circulating levels of CC16 [40]. Lower concentrations of CC16, an anti-inflammatory pneumoprotein produced by club cells in the airways, have been associated with lung function deficits [28, 51], increased airway resistance and hyperresponsiveness attributed to airways remodelling [51]. Furthermore, vitamin A treatment increased circulating CC16 in humans [27]. Thus, we speculate that an increase in CC16 might mediate the positive association between vitamin A intake and lung function. The interactions we found with CC16 support this hypothesis: higher vitamin A intake was associated with better lung function only in children with a genetic tendency to produce more CC16 (GG genotype) [40], suggesting that this genotype might carry a greater potential for upregulation by vitamin A.

The associations between preformed vitamin A intake and lung function measures were also modified by an *NCOR2* polymorphism. NCOR2 is in the retinoic acid signalling pathway and the variant is in a strong transcriptional enhancer element in lung fibroblasts [41]. The positive associations we found in carriers of the C allele, the variant associated with higher FVC in children [41], suggest that vitamin A might also have a role in lung growth through the regulation of fibroblasts. The negative associations seen in those homozygous for the T allele suggest a “flip-flop” gene–nutrient interaction [52].

Regarding *BCMO1* polymorphisms which influence vitamin A bioavailability, we hypothesised that children with genetically lower efficiency of β-carotene conversion may benefit more from a higher intake of preformed vitamin A. This was supported by the interactions with polymorphisms in the upstream *BCMO1* for both incident asthma and lung function. In contrast, when we stratified by a polymorphism in the coding region, an inverse association with incident asthma was paradoxically in high converters. Another unexpected finding was the positive association between carotene intake and incident asthma, when stratified by *BCMO1* genotypes. Given the contradictory and paradoxical nature of some of these gene–nutrient interactions, they should be interpreted with caution.

### Strengths and limitations

Strengths of the ALSPAC birth cohort include its population-based prospective design, large size, rich information on diet and potential confounders, and availability of the various genotype data. The post-bronchodilator assessment of lung function enabled us to better assess airway growth by eliminating reversible airflow limitation. We controlled for numerous potential confounders in the analyses and performed various sensitivity analyses; however, the possibility of unmeasured or residual confounding cannot be ruled out. A sizeable proportion of eligible children at 7 years were not included in our analyses, but our inverse probability weighting analysis showed that this is unlikely to have biased our findings, as generally expected in longitudinal studies [53]. While misclassification of the dietary exposures was inevitable, the prospective nature of the study makes them more likely to have been non-differential with respect to the outcomes, which would tend to bias effect estimates towards the null. Some other limitations of the FFQ include fewer items relevant to carotene intake compared with preformed vitamin A, and some important sources such as broccoli and sweet potato were not included. Given the semiquantitative nature of the FFQs, our estimated “absolute” intakes should be regarded as approximate. In view of the multiple analyses carried out, our main findings require replication. Given the *a priori* nature of the hypotheses, however, and the correlation between lung function measures, it did not seem appropriate to correct for multiple testing. However, findings with borderline statistical significance, such as the association with airflow limitation, should be interpreted cautiously. Finally, the generalisability of our findings to other populations, particularly those with an overt vitamin A deficiency, warrants further research.

### Conclusions

A higher intake of preformed vitamin A, but not β-carotene, in mid-childhood was associated with higher subsequent lung function and lower risk of fixed airflow limitation and incident asthma.

## Supplementary material

10.1183/13993003.04407-2020.Supp1 **Please note:** supplementary material is not edited by the Editorial Office, and is uploaded as it has been supplied by the author.
Supplementary material ERJ-04407-2020.SUPPLEMENT

## Shareable PDF

10.1183/13993003.04407-2020.Shareable1 This one-page PDF can be shared freely online.
Shareable PDF ERJ-04407-2020.Shareable

## References

[1] Timoneda J, Rodriguez-Fernandez L, Zaragoza R, et al. Vitamin A deficiency and the lung. Nutrients 2018; 10: 1132. doi:10.3390/nu10091132 PMC6164133 30134568

[2] Tang G. Bioconversion of dietary provitamin A carotenoids to vitamin A in humans. Am J Clin Nutr 2010; 91: 1468S–1473S. doi:10.3945/ajcn.2010.28674G 20200262 PMC2854912

[3] Massaro D, Massaro GD. Lung development, lung function, and retinoids. N Engl J Med 2010; 362: 1829–1831. doi:10.1056/NEJMe1002366 20463343

[4] Hind M, Gilthorpe A, Stinchcombe S, et al. Retinoid induction of alveolar regeneration: from mice to man? Thorax 2009; 64: 451–457. doi:10.1136/thx.2008.105437 19401491

[5] Marquez HA, Cardoso WV. Vitamin A-retinoid signaling in pulmonary development and disease. Mol Cell Pediatr 2016; 3: 28. doi:10.1186/s40348-016-0054-6 27480876 PMC4969253

[6] Schuster GU, Kenyon NJ, Stephensen CB. Vitamin A deficiency decreases and high dietary vitamin A increases disease severity in the mouse model of asthma. J Immunol 2008; 180: 1834–1842. doi:10.4049/jimmunol.180.3.1834 18209081

[7] Chen F, Marquez H, Kim YK, et al. Prenatal retinoid deficiency leads to airway hyperresponsiveness in adult mice. J Clin Invest 2014; 124: 801–811. doi:10.1172/JCI70291 24401276 PMC3904614

[8] Checkley W, West KP Jr, Wise RA, et al. Maternal vitamin A supplementation and lung function in offspring. N Engl J Med 2010; 362: 1784–1794. doi:10.1056/NEJMoa0907441 20463338

[9] Checkley W, West KP Jr, Wise RA, et al. Supplementation with vitamin A early in life and subsequent risk of asthma. Eur Respir J 2011; 38: 1310–1319. doi:10.1183/09031936.00006911 21700611 PMC7305825

[10] Parr CL, Magnus MC, Karlstad O, et al. Vitamin A and D intake in pregnancy, infant supplementation, and asthma development: the Norwegian Mother and Child Cohort. Am J Clin Nutr 2018; 107: 789–798. doi:10.1093/ajcn/nqy016 29722838 PMC6692651

[11] Allen S, Britton JR, Leonardi-Bee JA. Association between antioxidant vitamins and asthma outcome measures: systematic review and meta-analysis. Thorax 2009; 64: 610–619. doi:10.1136/thx.2008.101469 19406861

[12] Morabia A, Menkes MJ, Comstock GW, et al. Serum retinol and airway obstruction. Am J Epidemiol 1990; 132: 77–82. doi:10.1093/oxfordjournals.aje.a115645 2356816

[13] Mai XM, Langhammer A, Chen Y, et al. Cod liver oil intake and incidence of asthma in Norwegian adults – the HUNT study. Thorax 2013; 68: 25–30. doi:10.1136/thoraxjnl-2012-202061 22977130

[14] Agusti A, Noell G, Brugada J, et al. Lung function in early adulthood and health in later life: a transgenerational cohort analysis. Lancet Respir Med 2017; 5: 935–945. doi:10.1016/S2213-2600(17)30434-4 29150410

[15] Vasquez MM, Zhou M, Hu C, et al. Low lung function in young adult life is associated with early mortality. Am J Respir Crit Care Med 2017; 195: 1399–1401. doi:10.1164/rccm.201608-1561LE 28504596 PMC5443902

[16] Schultz ES, Hallberg J, Andersson N, et al. Early life determinants of lung function change from childhood to adolescence. Respir Med 2018; 139: 48–54. doi:10.1016/j.rmed.2018.04.009 29858001

[17] Martinez FD. Early-life origins of chronic obstructive pulmonary disease. N Engl J Med 2016; 375: 871–878. doi:10.1056/NEJMra1603287 27579637

[18] Belgrave DCM, Granell R, Turner SW, et al. Lung function trajectories from pre-school age to adulthood and their associations with early life factors: a retrospective analysis of three population-based birth cohort studies. Lancet Respir Med 2018; 6: 526–534. doi:10.1016/S2213-2600(18)30099-7 29628377

[19] Bui DS, Lodge CJ, Burgess JA, et al. Childhood predictors of lung function trajectories and future COPD risk: a prospective cohort study from the first to the sixth decade of life. Lancet Respir Med 2018; 6: 535–544. doi:10.1016/S2213-2600(18)30100-0 29628376

[20] Yammine S, Schmidt A, Sutter O, et al. Functional evidence for continued alveolarisation in former preterms at school age? Eur Respir J 2016; 47: 147–155. doi:10.1183/13993003.00478-2015 26493788

[21] Narayanan M, Owers-Bradley J, Beardsmore CS, et al. Alveolarization continues during childhood and adolescence: new evidence from helium-3 magnetic resonance. Am J Respir Crit Care Med 2012; 185: 186–191. doi:10.1164/rccm.201107-1348OC 22071328 PMC3410735

[22] Agusti A, Faner R. Lung function trajectories in health and disease. Lancet Respir Med 2019; 7: 358–364. doi:10.1016/S2213-2600(18)30529-0 30765254

[23] Guilleminault L, Williams EJ, Scott HA, et al. Diet and asthma: is it time to adapt our message? Nutrients 2017; 9; 1227. doi:10.3390/nu9111227 PMC5707699 29117118

[24] Julia V, Macia L, Dombrowicz D. The impact of diet on asthma and allergic diseases. Nat Rev Immunol 2015; 15: 308–322. doi:10.1038/nri3830 25907459

[25] Garcia-Larsen V, Ierodiakonou D, Jarrold K, et al. Diet during pregnancy and infancy and risk of allergic or autoimmune disease: a systematic review and meta-analysis. PLoS Med 2018; 15: e1002507. doi:10.1371/journal.pmed.1002507 29489823 PMC5830033

[26] Melen E, Guerra S. Recent advances in understanding lung function development. F1000Res 2017; 6: 726. doi:10.12688/f1000research.11185.1 28620467 PMC5461903

[27] Chen Y, Vasquez MM, Zhu L, et al. Effects of retinoids on augmentation of club cell secretory protein. Am J Respir Crit Care Med 2017; 196: 928–931. doi:10.1164/rccm.201608-1611LE 28231434 PMC5649971

[28] Guerra S, Halonen M, Vasquez MM, et al. Relation between circulating CC16 concentrations, lung function, and development of chronic obstructive pulmonary disease across the lifespan: a prospective study. Lancet Respir Med 2015; 3: 613–620. doi:10.1016/S2213-2600(15)00196-4 26159408 PMC4640928

[29] Boyd A, Golding J, Macleod J, et al. Cohort Profile: the ‘children of the 90s’ – the index offspring of the Avon Longitudinal Study of Parents and Children. Int J Epidemiol 2013; 42: 111–127. doi:10.1093/ije/dys064 22507743 PMC3600618

[30] Fraser A, Macdonald-Wallis C, Tilling K, et al. Cohort Profile: the Avon Longitudinal Study of Parents and Children: ALSPAC mothers cohort. Int J Epidemiol 2013; 42: 97–110. doi:10.1093/ije/dys066 22507742 PMC3600619

[31] Emmett P. Dietary assessment in the Avon Longitudinal Study of Parents and Children. Eur J Clin Nutr 2009; 63: Suppl. 1, S38–S44. doi:10.1038/ejcn.2008.63 19190642

[32] Ministry of Agriculture Fisheries and Food. Food Portion Sizes. London, HMSO, 1991.

[33] Holland B, Welch AA, Unwin ID, et al. McCance and Widdowson's The Composition of Foods. 5th Edn. London, Royal Society of Chemistry and Ministry of Agriculture, Fisheries and Food, 1991.

[34] Ministry of Agriculture Fisheries and Food. Fatty Acids: Supplement to McCance & Widdowson's The Composition of Foods. Cambridge, Royal Society of Chemistry, 1998.

[35] Quanjer PH, Stanojevic S, Stocks J, et al. Changes in the FEV_1_/FVC ratio during childhood and adolescence: an intercontinental study. Eur Respir J 2010; 36: 1391–1399. doi:10.1183/09031936.00164109 20351026

[36] Quanjer PH, Stanojevic S, Cole TJ, et al. Multi-ethnic reference values for spirometry for the 3–95-year age range: the global lung function 2012 equations. Eur Respir J 2012; 40: 1324–1343. doi:10.1183/09031936.00080312 22743675 PMC3786581

[37] Mahmoud O, Granell R, Tilling K, et al. Association of height growth in puberty with lung function: a longitudinal study. Am J Respir Crit Care Med 2018; 198: 1539–1548. doi:10.1164/rccm.201802-0274OC 29995435 PMC6298631

[38] American Thoracic Society. Standardization of spirometry, 1994 update. Am J Respir Crit Care Med 1995; 152: 1107–1136. doi:10.1164/ajrccm.152.3.7663792 7663792

[39] Cornish RP, Henderson J, Boyd AW, et al. Validating childhood asthma in an epidemiological study using linked electronic patient records. BMJ Open 2014; 4: e005345. doi:10.1136/bmjopen-2014-005345 PMC4010849 24760357

[40] Kim DK, Cho MH, Hersh CP, et al. Genome-wide association analysis of blood biomarkers in chronic obstructive pulmonary disease. Am J Respir Crit Care Med 2012; 186: 1238–1247. doi:10.1164/rccm.201206-1013OC 23144326 PMC3622441

[41] Minelli C, Dean CH, Hind M, et al. Association of forced vital capacity with the developmental gene *NCOR2*. PLoS One 2016; 11: e0147388. doi:10.1371/journal.pone.0147388 26836265 PMC4737618

[42] Borel P, Desmarchelier C. Genetic variations associated with vitamin A status and vitamin A bioavailability. Nutrients 2017; 9: 246. doi:10.3390/nu9030246 PMC5372909 28282870

[43] Ferrucci L, Perry JR, Matteini A, et al. Common variation in the beta-carotene 15,15′-monooxygenase 1 gene affects circulating levels of carotenoids: a genome-wide association study. Am J Hum Genet 2009; 84: 123–133. doi:10.1016/j.ajhg.2008.12.019 19185284 PMC2668002

[44] Lietz G, Oxley A, Leung W, et al. Single nucleotide polymorphisms upstream from the beta-carotene 15,15′-monoxygenase gene influence provitamin A conversion efficiency in female volunteers. J Nutr 2012; 142: 161S–165S. doi:10.3945/jn.111.140756 22113863

[45] Leung WC, Hessel S, Meplan C, et al. Two common single nucleotide polymorphisms in the gene encoding beta-carotene 15,15′-monoxygenase alter beta-carotene metabolism in female volunteers. FASEB J 2009; 23: 1041–1053. doi:10.1096/fj.08-121962 19103647

[46] Nurmatov U, Nwaru BI, Devereux G, et al. Confounding and effect modification in studies of diet and childhood asthma and allergies. Allergy 2012; 67: 1041–1059. doi:10.1111/j.1398-9995.2012.02858.x 22712878

[47] Textor J, van der Zander B, Gilthorpe MS, et al. Robust causal inference using directed acyclic graphs: the R package “dagitty”. Int J Epidemiol 2016; 45: 1887–1894. doi:10.1093/ije/dyw341 28089956

[48] Hernan MA, Hernandez-Diaz S, Robins JM. A structural approach to selection bias. Epidemiology 2004; 15: 615–625. doi:10.1097/01.ede.0000135174.63482.43 15308962

[49] Institute of Medicine Panel on Micronutrients. Dietary Reference Intakes for Vitamin A, Vitamin K, Arsenic, Boron, Chromium, Copper, Iodine, Iron, Manganese, Molybdenum, Nickel, Silicon, Vanadium, and Zinc Dietary Reference Intakes for Vitamin A, Vitamin K, Arsenic, Boron, Chromium, Copper, Iodine, Iron, Manganese, Molybdenum, Nickel, Silicon, Vanadium, and Zinc. Washington, The National Academies Press, 2001. 25057538

[50] Guenegou A, Leynaert B, Pin I, et al. Serum carotenoids, vitamins A and E, and 8 year lung function decline in a general population. Thorax 2006; 61: 320–326. doi:10.1136/thx.2005.047373 16565267 PMC2104600

[51] Zhai J, Insel M, Addison KJ, et al. Club cell secretory protein deficiency leads to altered lung function. Am J Respir Crit Care Med 2019; 199: 302–312. doi:10.1164/rccm.201807-1345OC 30543455 PMC6363971

[52] Ober C, Vercelli D. Gene-environment interactions in human disease: nuisance or opportunity? Trends Genet 2011; 27: 107–115. doi:10.1016/j.tig.2010.12.004 21216485 PMC3073697

[53] Howe LD, Tilling K, Galobardes B, et al. Loss to follow-up in cohort studies: bias in estimates of socioeconomic inequalities. Epidemiology 2013; 24: 1–9. doi:10.1097/EDE.0b013e31827623b1 23211345 PMC5102324

