# Supplementary material for: Dietary intake of vitamin A, lung function and incident asthma in childhood
Source: Eur Respir J. 2021 Oct 28;58(4):2004407. doi: 10.1183/13993003.04407-2020 (PMC8551559; doi:10.1183/13993003.04407-2020)
Supplement: Supplementary file 1 [file ERJ-04407-2020.SUPPLEMENT.pdf]

## Online supplementary materials

**Dietary intake of vitamin A, lung function, and incident asthma in childhood**

Mohammad Talaei, David A Hughes, Osama Mahmoud, Pauline M. Emmett, Raquel Granell, Stefano Guerra, Seif O. Shaheen

**Corresponding author:**

Dr Mohammad Talaei, Institute of Population Health Sciences, Barts and The London School of Medicine and Dentistry, 58 Turner Street, London, E1 2AB, UK. E-mail: mohammad.talaei@u.nus.edu; Tel: +44(0)20 7882 2499

## Contents

**Further details** .......... **3**

Information on covariates .......... 3

Genotyping .......... 4

Multivariable models .......... 5

Sensitivity analyses .......... 5

Restricted cubic spline analysis .......... 6

Inverse probability weighting .......... 6

**References** .......... **7**

**Supplementary tables and figures** .......... **8**

**Supplementary Figure E1.** Study profile. .......... 8

**Supplementary Figure E2.** Directed acyclic graph to study covariates and potential structural confounding bias for the association between child's vitamin A intake and lung function. .......... 9

**Supplementary Figure E3.** Median intake in quartiles of preformed vitamin A and retinol activity equivalent (RAE) at 7 years of age in ALSPAC in relation to recommended intake (Recommended Dietary Allowance [4]) .......... 10

**Supplementary Table E1.** Characteristics of selected single-nucleotide polymorphisms .......... 11

**Supplementary Table E2:** Participant* characteristics according to quartiles of β-carotene equivalent intake at 7 years of age .......... 12

**Supplementary Table E3:** Linear regression coefficients (95% confidence interval) for pre-bronchodilator lung function measures (z scores) according to quartiles of intakes of preformed vitamin A and β-carotene equivalent, adjusted for potential confounders .......... 14

**Supplementary Table E4:** Linear regression coefficients (95% confidence interval) for post-bronchodilator lung function measures (z scores) according to quartiles of intakes of preformed vitamin A and β-carotene equivalent, stratified by *BCMO1* genotype (coding region SNPs) .......... 15

**Supplementary Table E5:** Odds ratio (95% confidence interval) for incident asthma at 11 or 14 years according to quartiles of intakes of preformed vitamin A and β-carotene equivalent, stratified by other *BCMO1* genotypes .......... 17

**Supplementary Table E6:** Linear regression coefficients (95% confidence interval) for post-bronchodilator lung function measures (z scores) according to quartiles of intakes of preformed vitamin A, adjusted for further potential confounders .......... 19

**Supplementary Table E7:** Linear regression coefficients (95% confidence interval) for post-bronchodilator lung function measures (z scores) according to quartiles of intakes of β-carotene equivalent, adjusted for further potential confounders .......... 21

**Supplementary Table E8:** Odds ratio (95% confidence interval) for incident asthma at 11 or 14 years according to quartiles of intakes of preformed vitamin A and β-carotene equivalent, adjusted for further potential confounders .......... 23

**Supplementary Table E9:** Associations of energy adjusted intakes of preformed vitamin A and β-carotene equivalent using residual method with incident asthma at 11 or 14 years and post-bronchodilator lung function measures (z scores), adjusted for potential confounders .......... 24

## Further details

### Information on covariates

Living location was defined based on the 2001 Census urban/rural indicator at 7 years of age. A maternal history of hay fever, asthma, and eczema was ascertained at 12 weeks of gestation, and any positive response was considered as a maternal history of atopic disease. Paternal history of atopic disease was defined similarly through questions asked about partners during pregnancy or early after delivery. Mothers were asked how many cigarettes they smoked per day when the child was 7 years of age. We defined childhood food allergy if there was any such report by mothers at 6 (to milk), 30, 54, or 81 months of age. Data on maternal ethnicity and indicators of socioeconomic status (maternal education, housing tenure and financial difficulty in pregnancy) were collected at various time points during pregnancy (8, 18, and 32 weeks of gestation) and at 8 weeks postpartum. Number of older and younger siblings was asked at 7 years; if data were missing, we used data on parity to calculate the number of older siblings. Child atopy was defined by a positive reaction (maximum diameter of any detectable weal) to Dermatophagoides pteronyssinus, cat or grass (after subtracting positive saline reactions from histamine and allergen weals, and excluding children unreactive to 1% histamine) at 7 years. We used information on supplement use collected at 78 months of age and defined overall use, as well as supplements containing vitamin A. Three separate dietary patterns, 'health-conscious', 'traditional', and 'junk', were previously defined using principal component analysis. The health-conscious and traditional patterns were associated with better nutrient profiles than the processed pattern (junk) which tended to be energy-dense and nutrient-poor [1]. Frequency of child's participation in vigorous physical activity (such as running, dance, gymnastics, netball, swimming, or aerobics) during the past month was asked at 8 years of age.

Child's body mass index was calculated as weight (kg) divided by height squared ($m^2$), measured at age 7 years. BMI was missing for around 12% of participants included in these analyses. We used a forward stepwise logistic regression analysis to define a model that predicts BMI. Among potential variables initially included, 11 factors significantly contributed to the model (sex, total energy intake, vigorous physical activity, older siblings, younger siblings, any supplement use, season of data collection, maternal education, maternal history of atopy, financial difficulty during pregnancy, maternal smoking at 7 years).

We applied this model to impute missing BMI using the corresponding coefficients of these factors. The mean ± SD of BMI was 16.2 ± 2.1 kg/m$^2$ originally and 16.2 ± 1.9 kg/m$^2$ after imputation.

**Genotyping**

The majority of the children's DNA samples were extracted from cord blood or venous blood collected at age 7 years, with a small number extracted from venous blood collected at 43–61 months. ALSPAC children were genotyped using the Illumina HumanHap550 quad chip genotyping platforms by 23andme subcontracting the Wellcome Trust Sanger Institute, Cambridge, UK and the Laboratory Corporation of America, Burlington, NC, US. The resulting raw genome-wide data were subjected to standard quality control methods. Individuals were excluded on the basis of gender mismatches; minimal or excessive heterozygosity; disproportionate levels of individual missingness (>3%) and insufficient sample replication (IBD < 0.8). Population stratification was assessed by multidimensional scaling analysis and compared with Hapmap II (release 22) European descent (CEU), Han Chinese, Japanese and Yoruba reference populations; all individuals with non-European ancestry were removed. SNPs with a minor allele frequency of < 1%, a call rate of < 95% or evidence for violations of Hardy-Weinberg equilibrium (P < 5E-7) were removed. Cryptic relatedness was measured as proportion of identity by descent (IBD > 0.1). Related subjects that passed all other quality control thresholds were retained during subsequent phasing and imputation. 9,115 subjects and 500,527 SNPs passed these quality control filters.

We combined 477,482 SNP genotypes in common between the sample of mothers and sample of children. We removed SNPs with genotype missingness above 1% due to poor quality (11,396 SNPs removed) and removed a further 321 subjects due to potential ID mismatches. This resulted in a dataset of 17,842 subjects containing 6,305 duos and 465,740 SNPs (112 were removed during liftover and 234 were out of HWE after combination). We estimated haplotypes using ShapeIT (v2.r644) which utilises relatedness during phasing. Imputation was performed using IMPUTE2 and the HRC reference panel (v1.1). Table E1 shows the SNPs included in this analysis. None were in linkage disequilibrium; the largest $R^2$ value among these SNPs was 0.74, between rs6564851 and rs6420424, all other $R^2$ values were lower than 0.52.

**Multivariable models**

In the multivariable models, we first adjusted for sex and total energy intake (kJ·day−1) at 7 years. The second model additionally included maternal ethnicity (white, non-white) and three indicators of socioeconomic status, namely, maternal education (secondary education, vocational, O level, A level, degree, and missing), housing tenure during pregnancy (mortgaged/owned, council rented, non-council rented, unknown/missing), and financial difficulty during pregnancy (yes/no), maternal history of atopic disease (yes/no), paternal history of atopic disease (yes/no), maternal smoking when the child was 7 years of age (none, 1-9, 10-19, and ≥20/day), older sibling (yes/no), younger sibling (yes/no), any use of supplements (yes/no), and season when the FFQ was completed (winter, spring, summer, autumn). Data on potential confounders in multivariable models were missing for 4.2% at most and included in the analyses as separate 'missing' categories.

**Sensitivity analyses**

The sensitivity of our findings to adjustment for other potential confounders was tested by further adjusting for dietary patterns ('health-conscious', 'junk', and 'traditional', separately) score as quartiles at 7 years, breastfeeding by the 3rd month (never, stopped/non-exclusive, exclusive), any history of food allergy (binary), living location (urban vs. rural), vigorous physical activity (none or less than once a week, 1-3 times a week, 4-6 times a week, and daily), BMI (continuous) at 7 years, atopy (binary), and maternal intake of preformed vitamin A and carotene at 32 weeks of gestation (quartiles). In another model, we further adjusted for dietary intake of vitamin C, vitamin D, vitamin E, zinc, omega-3 from fish, and total protein (all as quartiles) as potential confounders. In a separate model, we also mutually adjusted preformed vitamin A and carotene intakes. The associations of our exposure variables with pre-bronchodilator lung function measures were also tested. We used the residual method [2] to further adjust dietary intakes of preformed vitamin A and carotene for total energy intake and examined the new adjusted variables in the same multivariable models.

We also explored the impact of excluding children of non-white mothers, with any history of food allergy before 7 years of age, with an extreme total energy intake above the 95$^{th}$ percentile or below the 5$^{th}$

percentile, with asthma at 7 years of age (for lung function measures), with asthma at 14 years of age (for lung function measures), and those who consumed vitamin A containing supplements (in separate models).

**Restricted cubic spline analysis**

Restricted cubic spline analysis was used to examine the shape of relationship between sources of vitamin A intakes (preformed and carotene) and lung function measures and asthma in multivariable-adjusted models. We selected the number of knots based on the values of Akaike information criteria (AIC) to fit the best-approximating model, chose the first knot as reference, and tested for linearity by the Wald-test.

**Inverse probability weighting**

Inverse probability weighting is a technique to correct for selection bias [3]. In a two-step method, the probability of selection in the study is estimated for everyone based on a given set of covariates and exposure; then the inverse of this probability is included in the analysis as a weight. Inverse probability weighting creates a pseudo-population in which each selected subject accounts for those with similar characteristics who were not selected.

Accordingly, among 7,183 children with data on vitamin A intake who were not diagnosed with current asthma at 7 years, we estimated the probability of selection of 4,540 children for given values of covariates using a logistic regression model. Unselected children were those of unknown asthma status at 7, 11, or 14 years. Similarly, we estimated the probability of selection of 2,985 children with data on all lung function measures at 15.5 years for given values of covariates among 8,135 children with data on vitamin A intake. These covariates included all factors in model 2 (namely, sex, total energy intake, maternal education, housing tenure during pregnancy, financial difficulty during pregnancy, maternal ethnicity, maternal history of atopic disease, maternal age, maternal smoking, older sibling, younger sibling, and season of dietary data collection), plus quartiles of preformed vitamin A and carotene intake, quartiles of health-conscious dietary pattern score, and history of food allergy. Then, we assigned the inverse of this probability as the weight for each participant, and carried out a multivariable weighted logistic or linear regression analysis to test the associations of quartiles of preformed vitamin A and carotene intake with incident asthma or lung function measures in a pseudo-population, which, in contrast to the selected

population, is unaffected by selection bias due to these factors. In other words, this approach tests if the observed associations in the main analysis were sensitive to unknown asthma status at baseline (for incident asthma) or loss to follow-up (for both lung function measures and incident asthma).

## References


1. Emmett PM, Jones LR, Northstone K. Dietary patterns in the Avon Longitudinal Study of Parents and Children. *Nutr Rev* 2015: 73 Suppl 3: 207-230.

2. Willett WC, Howe GR, Kushi LH. Adjustment for total energy intake in epidemiologic studies. *Am J Clin Nutr* 1997: 65(4 Suppl): 1220S-1228S; discussion 1229S-1231S.

3. Hernan MA, Hernandez-Diaz S, Robins JM. A structural approach to selection bias. *Epidemiology* 2004: 15(5): 615-625.

4. Institute of Medicine. Dietary Reference Intakes for Vitamin A, Vitamin K, Arsenic, Boron, Chromium, Copper, Iodine, Iron, Manganese, Molybdenum, Nickel, Silicon, Vanadium, and Zinc Dietary Reference Intakes for Vitamin A, Vitamin K, Arsenic, Boron, Chromium, Copper, Iodine, Iron, Manganese, Molybdenum, Nickel, Silicon, Vanadium, and Zinc. The National Academies Press, Washington (DC), 2001.

## Supplementary tables and figures

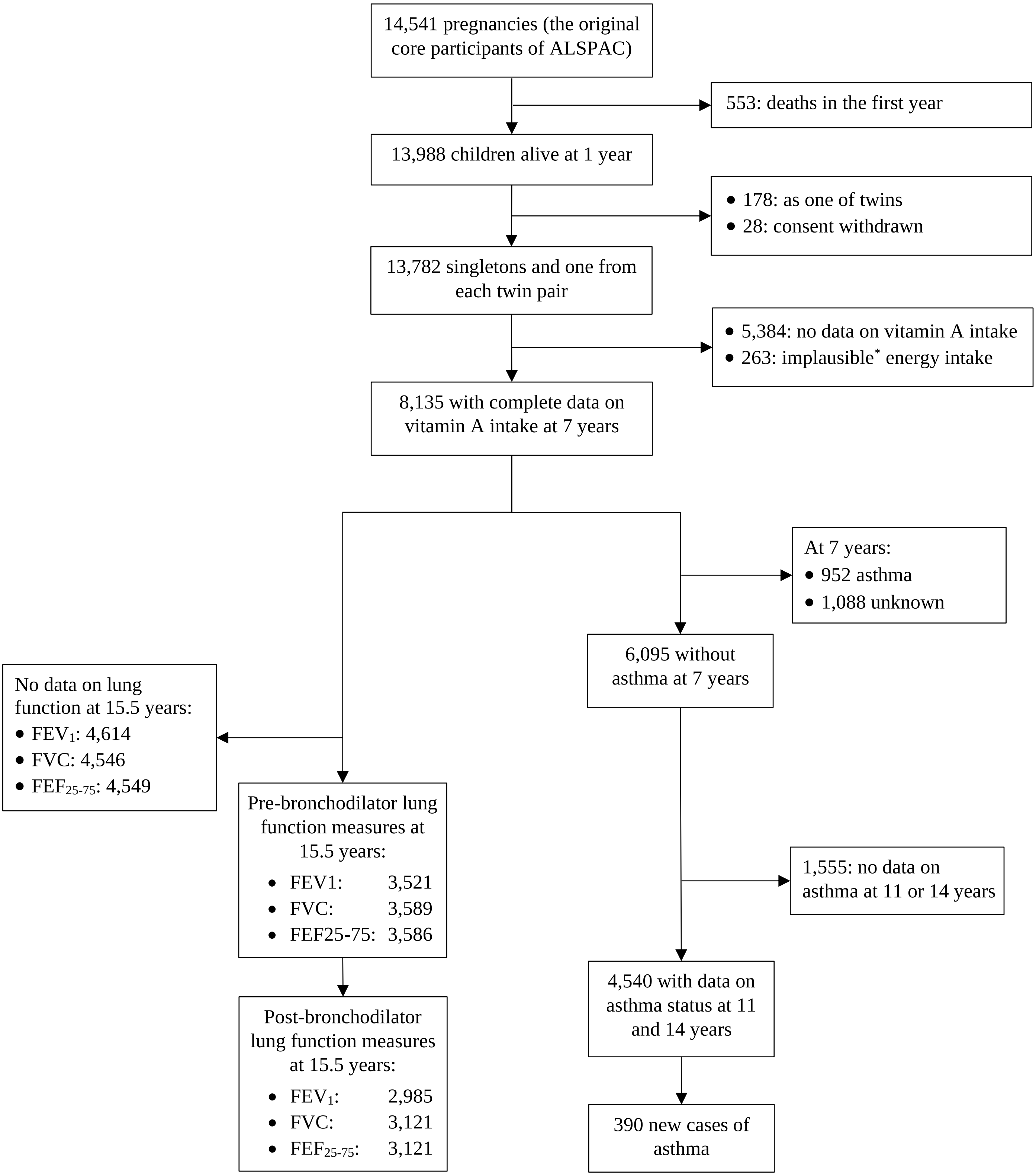


**Supplementary Figure E1.** Study profile.

* Weekly total energy intake of <15000 kJ or >140000 kJ

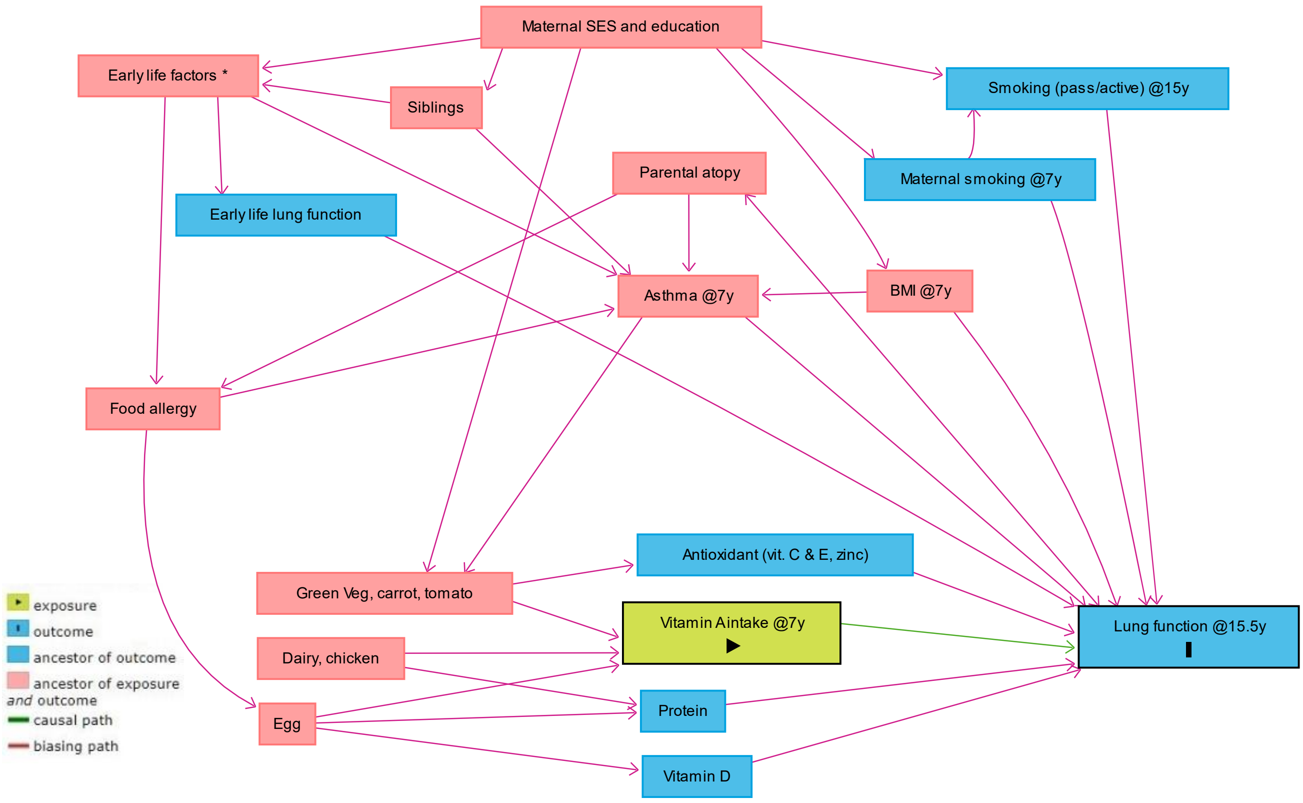


**Supplementary Figure E2.** Directed acyclic graph to study covariates and potential structural confounding bias for the association between child's vitamin A intake and lung function.

* Early life factors: Maternal smoking in pregnancy/infancy, gestational age, birth season, lower respiratory tract infection in infancy, day care, breastfeeding.

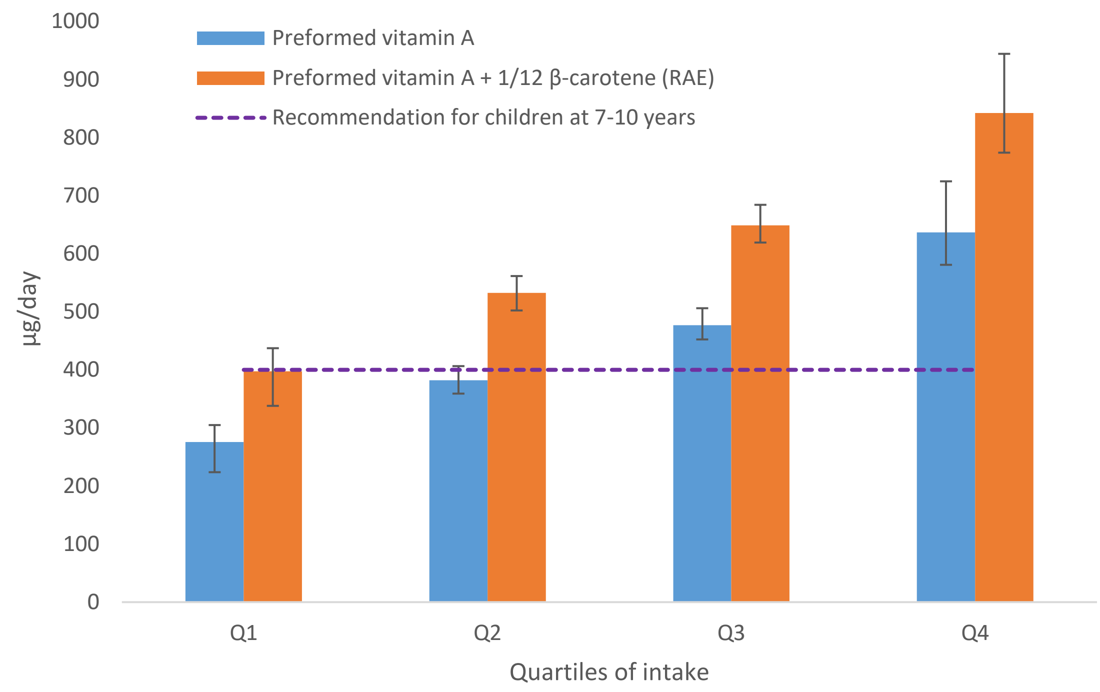


**Supplementary Figure E3.** Median intake in quartiles of preformed vitamin A and retinol activity equivalent (RAE) at 7 years of age in ALSPAC in relation to recommended intake (Recommended Dietary Allowance [4])

Whiskers show interquartile range.

**Supplementary Table E1.** Characteristics of selected single-nucleotide polymorphisms

| rs number | Chr. | Position (GRCh38) | Gene | Location | Alleles | MAF* | Effect allele |
|---|---|---|---|---|---|---|---|
| rs7501331 | 16 | 81280891 | *BCMO1* | Coding region | C, T | 0.23 (T) | C† |
| rs12934922 | 16 | 81268089 | *BCMO1* | Coding region | A, T | 0.44 (T) | A† |
| rs6564851 | 16 | 81230992 | near *BCMO1* | Upstream | T, G | 0.47 (T) | T† |
| rs11645428 | 16 | 81225291 | near *BCMO1* | Upstream | A, G | 0.33 (A) | A† |
| rs6420424 | 16 | 81208497 | *PKD1L2* | Upstream** | A, G | 0.48 (A) | G† |
| rs3741240 | 11 | 62419070 | *SCGB1A1* | 5' UTR | A, G | 0.35 (A) | G†† |
| rs12708369 | 12 | 124391031 | *NCOR2* | Intron | C, T | 0.39 (T) | C‡ |

Chr: Chromosome; *BCMO1*: β-Carotene 15,15'-monooxygenase (also called *BCO1*); *PKD1L2*: polycystic kidney disease protein 1-like 2; *SCGB1A1*: Secretoglobin Family 1A Member 1 (Club cell secretory protein coding gene); *NCOR2*: nuclear receptor corepressor 2

* Based on frequency in ALSPAC population (https://www.ncbi.nlm.nih.gov/snp)

** Upstream from the *BCMO1* gene

† High efficiency in conversion of β-carotene provitamin A

†† High serum level of CC16 (Club cell secretory protein)

‡ Associated with increased FVC

**Supplementary Table E2:** Participant* characteristics according to quartiles of β-carotene equivalent intake at 7 years of age

| | Quartiles of β-carotene intake | | | | |
|---|---|---|---|---|---|
| | **Q1** | **Q2** | **Q3** | **Q4** | **P-value** |
| n (%) | 1317 (24.5) | 1345 (25.0) | 1382 (25.7) | 1340 (24.9) | |
| β-carotene intake intake, μg/d | 961 ± 362 | 1603 ± 79.5 | 1971 ± 164 | 3262 ± 607 | |
| Male, n (%) | 684 (51.9) | 668 (49.7) | 640 (46.3) | 667 (49.8) | 0.03 |
| Older siblings, n (%) | 665 (50.5) | 736 (54.7) | 690 (49.9) | 696 (51.9) | 0.06 |
| Younger siblings, n (%) | 668 (50.7) | 674 (50.1) | 738 (53.4) | 717 (53.5) | 0.17 |
| Total energy intake, kJ/day | 6666 ± 1629 | 7100 ± 1306 | 8123 ± 1483 | 8433 ± 1883 | <0.001 |
| BMI, kg/m$^2$ | 16.2 ± 2.1 | 16.1 ± 1.9 | 16.2 ± 2.0 | 16.1 ± 1.8 | 0.40 |
| Health conscious dietary pattern score | -0.32 ± 0.82 | -0.12 ± 0.86 | 0.09 ± 0.92 | 0.38 ± 1.10 | <0.001 |
| Season of dietary information collection, n (%) | | | | | 0.76 |
| Winter | 327 (24.8) | 342 (25.4) | 368 (26.6) | 342 (25.5) | |
| Spring | 403 (30.6) | 402 (29.9) | 389 (28.1) | 391 (29.2) | |
| Summer | 386 (29.3) | 358 (26.6) | 395 (28.6) | 376 (28.1) | |
| Autumn | 187 (14.2) | 230 (17.1) | 214 (15.5) | 217 (16.2) | |
| Missing | 14 (1.1) | 13 (1.0) | 16 (1.2) | 14 (1.0) | |
| History of food allergy, n (%) | 236 (17.9) | 212 (15.8) | 237 (17.1) | 252 (18.8) | 0.20 |
| Any supplement use, n (%) | 449 (34.1) | 426 (31.7) | 448 (32.4) | 481 (35.9) | 0.09 |
| Protein intake, g/d | 55.1 ± 14.2 | 60.6 ± 11.2 | 68.8 ± 13.5 | 72.6 ± 16.9 | <0.001 |
| Vitamin C intake, mg/d | 59.2 ± 30.1 | 69.2 ± 28.4 | 81.6 ± 30.8 | 94.0 ± 35.0 | <0.001 |
| Vitamin D intake, mg/d | 2.47 ± 1.0 | 2.69 ± 0.8 | 3.05 ± 0.9 | 3.05 ± 1.1 | <0.001 |
| Vitamin E intake, mg/d | 8.57 ± 3.7 | 9.18 ± 3.2 | 10.36 ± 3.3 | 10.68 ± 3.8 | <0.001 |
| Zinc intake, mg/d | 5.28 ± 1.5 | 5.95 ± 1.2 | 6.81 ± 1.4 | 7.31 ± 1.8 | <0.001 |
| Total n-3 intake from fish, (mg/d) | 60.7 ± 68.8 | 75.8 ± 74.9 | 93.1 ± 96.6 | 95.7 ± 103.0 | <0.001 |
| **Parental factors** | | | | | |
| Maternal age at pregnancy, year | 29.3 ± 4.4 | 29.2 ± 4.4 | 29.3 ± 4.5 | 29.7 ± 4.3 | 0.01 |
| Maternal education, n (%) | | | | | <0.001 |
| Secondary or vocational | 302 (22.9) | 283 (21.0) | 238 (17.2) | 198 (14.8) | |
| O level | 471 (35.8) | 475 (35.3) | 481 (34.8) | 414 (30.9) | |
| A level or degree | 524 (39.8) | 567 (42.2) | 643 (46.5) | 710 (53.0) | |
| Missing | 20 (1.5) | 20 (1.5) | 20 (1.4) | 18 (1.3) | |
| Housing tenure during pregnancy, n (%) | | | | | 0.07 |
| Mortgaged/owned | 1075 (81.6) | 1126 (83.7) | 1162 (84.1) | 1151 (85.9) | |

| | | | | | |
|---|---|---|---|---|---|
| Council rented | 93 (7.1) | 83 (6.2) | 81 (5.9) | 69 (5.1) | |
| Non-council rented | 86 (6.5) | 66 (4.9) | 75 (5.4) | 77 (5.7) | |
| Missing | 63 (4.8) | 70 (5.2) | 64 (4.6) | 43 (3.2) | |
| Financial difficulty, n (%) | | | | | 0.41 |
| No | 1109 (84.2) | 1156 (85.9) | 1164 (84.2) | 1121 (83.7) | |
| Yes | 204 (15.5) | 181 (13.5) | 208 (15.1) | 213 (15.9) | |
| Missing | 4 (0.3) | 8 (0.6) | 10 (0.7) | 6 (0.4) | |
| Maternal ethnicity, n (%) | | | | | 0.39 |
| White | 1271 (96.5) | 1305 (97.0) | 1344 (97.3) | 1300 (97.0) | |
| Non-white | 25 (1.9) | 13 (1.0) | 18 (1.3) | 21 (1.6) | |
| Missing | 21 (1.6) | 27 (2.0) | 20 (1.4) | 19 (1.4) | |
| Maternal history of atopy, n (%) | | | | | 0.95 |
| No | 696 (52.8) | 689 (51.2) | 707 (51.2) | 685 (51.1) | |
| Yes | 571 (43.4) | 605 (45.0) | 623 (45.1) | 609 (45.4) | |
| Missing | 50 (3.8) | 51 (3.8) | 52 (3.8) | 46 (3.4) | |
| Paternal history of atopy, n (%) | | | | | 0.74 |
| No | 559 (42.4) | 565 (42.0) | 604 (43.7) | 592 (44.2) | |
| Yes | 419 (31.8) | 426 (31.7) | 405 (29.3) | 407 (30.4) | |
| Missing | 339 (25.7) | 354 (26.3) | 373 (27.0) | 341 (25.4) | |
| Maternal smoking, n (%) | | | | | 0.32 |
| No | 1031 (78.3) | 1098 (81.6) | 1093 (79.1) | 1085 (81.0) | |
| Yes | 235 (17.8) | 202 (15.0) | 231 (16.7) | 203 (15.1) | |
| Missing | 51 (3.9) | 45 (3.3) | 58 (4.2) | 52 (3.9) | |
| β-carotene intake at 32w of gestation, µg/d | 1892 ± 1031 | 2088 ± 1044 | 2216 ± 1176 | 2616 ± 1330 | <0.001 |

* Children included in incident asthma or lung function analysis (n= 5,384).

Numbers are mean ± SD unless otherwise specified.

**Supplementary Table E3:** Linear regression coefficients (95% confidence interval) for pre-bronchodilator lung function measures (z scores) according to quartiles of intakes of preformed vitamin A and β-carotene equivalent, adjusted for potential confounders

| | Quartiles of vitamin A intake | | | | P for | Per SD |
|---|---|---|---|---|---|---|
| | **Q1** | **Q2** | **Q3** | **Q4** | **trend*** | |
| **Preformed vitamin A** | | | | | | |
| Median (IQR), mg/d | 276 (224-305) | 382 (359-407) | 477 (452-506) | 637 (581-721) | | |
| **$FEV_1$** | | | | | | |
| Model 1 | 0.00 | 0.02 (-0.10, 0.14) | 0.06 (-0.06, 0.19) | 0.17 (0.02, 0.32) | 0.017 | 0.05 (-0.01, 0.10) |
| Model 2 | 0.00 | 0.02 (-0.10, 0.15) | 0.07 (-0.06, 0.20) | 0.19 (0.04, 0.34) | 0.010 | 0.05 (-0.01, 0.11) |
| **FVC** | | | | | | |
| Model 1 | 0.00 | -0.01 (-0.14, 0.11) | -0.04 (-0.16, 0.09) | 0.10 (-0.04, 0.25) | 0.15 | 0.02 (-0.04, 0.07) |
| Model 2 | 0.00 | -0.01 (-0.13, 0.11) | -0.03 (-0.16, 0.10) | 0.11 (-0.03, 0.26) | 0.12 | 0.02 (-0.04, 0.07) |
| **$FEV_1$/FVC ratio** | | | | | | |
| Model 1 | 0.00 | 0.06 (-0.05, 0.18) | 0.15 (0.03, 0.27) | 0.11 (-0.03, 0.25) | 0.11 | 0.03 (-0.02, 0.09) |
| Model 2 | 0.00 | 0.06 (-0.06, 0.18) | 0.14 (0.02, 0.26) | 0.12 (-0.02, 0.26) | 0.08 | 0.04 (-0.02, 0.09) |
| **$FEF_{25–75}$** | | | | | | |
| Model 1 | 0.00 | 0.06 (-0.05, 0.17) | 0.11 (-0.00, 0.23) | 0.15 (0.02, 0.29) | 0.017 | 0.05 (-0.00, 0.10) |
| Model 2 | 0.00 | 0.06 (-0.05, 0.17) | 0.11 (-0.00, 0.23) | 0.17 (0.04, 0.30) | 0.010 | 0.05 (0.00, 0.10) |
| **β-carotene equivalent** | | | | | | |
| Median (IQR), mg/d | 956 (646-1328) | 1607 (1538-1671) | 1945 (1827-2105) | 3268 (2670-3616) | | |
| **$FEV_1$** | | | | | | |
| Model 1 | 0.00 | 0.06 (-0.06, 0.18) | 0.10 (-0.03, 0.22) | 0.02 (-0.11, 0.15) | 0.91 | 0.02 (-0.03, 0.06) |
| Model 2 | 0.00 | 0.06 (-0.06, 0.18) | 0.11 (-0.02, 0.23) | 0.03 (-0.11, 0.16) | 0.98 | 0.02 (-0.03, 0.07) |
| **FVC** | | | | | | |
| Model 1 | 0.00 | 0.04 (-0.08, 0.16) | 0.04 (-0.09, 0.16) | -0.01 (-0.13, 0.12) | 0.72 | 0.01 (-0.03, 0.06) |
| Model 2 | 0.00 | 0.04 (-0.08, 0.16) | 0.05 (-0.08, 0.17) | 0.00 (-0.12, 0.13) | 0.86 | 0.02 (-0.03, 0.06) |
| **$FEV_1$/FVC ratio** | | | | | | |
| Model 1 | 0.00 | 0.03 (-0.08, 0.15) | 0.06 (-0.06, 0.18) | 0.00 (-0.12, 0.12) | 0.80 | -0.01 (-0.05, 0.03) |
| Model 2 | 0.00 | 0.02 (-0.09, 0.14) | 0.06 (-0.06, 0.18) | -0.01 (-0.13, 0.12) | 0.72 | -0.01 (-0.06, 0.03) |
| **$FEF_{25–75}$** | | | | | | |
| Model 1 | 0.00 | 0.05 (-0.05, 0.16) | 0.09 (-0.02, 0.20) | 0.01 (-0.10, 0.13) | 0.85 | 0.00 (-0.04, 0.04) |
| Model 2 | 0.00 | 0.04 (-0.07, 0.15) | 0.08 (-0.03, 0.20) | 0.00 (-0.11, 0.12) | 0.76 | -0.00 (-0.04, 0.04) |

$FEV_1$: forced expiratory volume in 1s; FVC: forced vital capacity; $FEF_{25–75}$: forced expiratory flow at 25–75% of FVC

* Linear trend was tested by treating the median values of quartiles as a continuous variable

Multivariable model 1: sex and total energy intake;

Multivariable model 2: further adjusted for maternal education, housing tenure at birth, financial difficulty during pregnancy, maternal ethnicity, maternal history of atopic disease, paternal history of atopic disease, maternal smoking, older sibling, younger sibling, supplement use, and season when the FFQ was completed.

**Supplementary Table E4:** Linear regression coefficients (95% confidence interval) for post-bronchodilator lung function measures (z scores) according to quartiles of intakes of preformed vitamin A and β-carotene equivalent, stratified by *BCMO1* genotype (coding region SNPs)

| | Quartiles of vitamin A intake | | | | P for | P for |
|---|---|---|---|---|---|---|
| | Q1 | Q2 | Q3 | Q4 | trend* | interaction |
| **Preformed vitamin A** | | | | | | |
| ***BCMO1* coding region: rs7501331** | | | | | | |
| **$FEV_1$** | | | | | | |
| CC† | 0.00 | -0.09 (-0.29, 0.11) | -0.03 (-0.24, 0.17) | 0.03 (-0.20, 0.27) | 0.60 | |
| CT | 0.00 | 0.08 (-0.17, 0.32) | 0.00 (-0.25, 0.26) | 0.32 (0.02, 0.63) | 0.04 | 0.41 |
| TT | 0.00 | -0.33 (-0.94, 0.29) | 0.15 (-0.52, 0.83) | 0.69 (-0.14, 1.52) | 0.08 | 0.03 |
| **FVC** | | | | | | |
| CC† | 0.00 | -0.03 (-0.23, 0.16) | -0.05 (-0.25, 0.15) | -0.02 (-0.24, 0.21) | 0.91 | |
| CT | 0.00 | 0.06 (-0.17, 0.30) | 0.03 (-0.22, 0.27) | 0.24 (-0.05, 0.52) | 0.12 | 0.37 |
| TT | 0.00 | -0.26 (-0.83, 0.31) | 0.10 (-0.54, 0.74) | 0.87 (0.09, 1.66) | 0.04 | 0.01 |
| **$FEV_1$/FVC ratio** | | | | | | |
| CC† | 0.00 | -0.08 (-0.24, 0.09) | 0.03 (-0.14, 0.21) | 0.08 (-0.12, 0.28) | 0.25 | |
| CT | 0.00 | 0.07 (-0.15, 0.29) | 0.04 (-0.19, 0.27) | 0.13 (-0.14, 0.40) | 0.39 | 0.89 |
| TT | 0.00 | 0.15 (-0.40, 0.70) | 0.52 (-0.08, 1.12) | 0.15 (-0.59, 0.89) | 0.48 | 0.89 |
| **$FEF_{25–75}$** | | | | | | |
| CC† | 0.00 | -0.10 (-0.27, 0.07) | -0.04 (-0.21, 0.14) | 0.06 (-0.13, 0.26) | 0.35 | |
| CT | 0.00 | 0.13 (-0.09, 0.36) | 0.10 (-0.13, 0.33) | 0.25 (-0.02, 0.52) | 0.09 | 0.64 |
| TT | 0.00 | 0.02 (-0.53, 0.58) | 0.27 (-0.35, 0.89) | 0.29 (-0.46, 1.05) | 0.36 | 0.33 |
| **β-carotene equivalent** | | | | | | |
| ***BCMO1* coding region: rs12934922** | | | | | | |
| **$FEV_1$** | | | | | | |
| AA† | 0.00 | -0.13 (-0.39, 0.13) | 0.08 (-0.18, 0.34) | 0.25 (-0.03, 0.52) | 0.02 | |
| AT | 0.00 | 0.12 (-0.09, 0.33) | 0.09 (-0.14, 0.31) | -0.12 (-0.35, 0.11) | 0.15 | 0.01 |
| TT | 0.00 | 0.12 (-0.25, 0.48) | 0.32 (-0.05, 0.68) | 0.06 (-0.32, 0.43) | 0.99 | 0.12 |
| **FVC** | | | | | | |
| AA† | 0.00 | -0.15 (-0.40, 0.10) | 0.03 (-0.22, 0.28) | 0.20 (-0.06, 0.46) | 0.04 | |
| AT | 0.00 | 0.04 (-0.16, 0.24) | 0.04 (-0.17, 0.26) | -0.10 (-0.33, 0.12) | 0.24 | 0.04 |
| TT | 0.00 | 0.12 (-0.22, 0.46) | 0.28 (-0.06, 0.62) | -0.02 (-0.37, 0.33) | 0.66 | 0.12 |
| **$FEV_1$/FVC ratio** | | | | | | |
| AA† | 0.00 | 0.11 (-0.12, 0.34) | 0.09 (-0.14, 0.32) | 0.03 (-0.21, 0.28) | 0.96 | |
| AT | 0.00 | 0.11 (-0.06, 0.29) | -0.01 (-0.20, 0.18) | -0.12 (-0.32, 0.07) | 0.10 | 0.25 |
| TT | 0.00 | -0.06 (-0.36, 0.24) | 0.03 (-0.27, 0.33) | 0.09 (-0.22, 0.40) | 0.45 | 0.85 |
| **$FEF_{25–75}$** | | | | | | |
| AA† | 0.00 | -0.07 (-0.30, 0.15) | 0.01 (-0.23, 0.24) | 0.16 (-0.08, 0.40) | 0.09 | |
| AT | 0.00 | 0.18 (-0.00, 0.35) | 0.11 (-0.08, 0.30) | -0.11 (-0.31, 0.08) | 0.07 | 0.01 |
| TT | 0.00 | 0.05 (-0.26, 0.37) | 0.26 (-0.05, 0.57) | 0.28 (-0.04, 0.60) | 0.08 | 0.82 |

$FEV_1$: forced expiratory volume in 1s; FVC: forced vital capacity; $FEF_{25–75}$: forced expiratory flow at 25–75% of FVC; *BCMO1*: β-carotene 15,15'-monooxygenase

* Linear trend was tested by treating the median values of quartiles as a continuous variable

† Homozygous alleles linked to a more efficient conversion of carotene provitamin A
Multivariable model: sex, total energy intake, maternal education, housing tenure at birth, financial difficulty during pregnancy, maternal ethnicity, maternal history of atopic disease, paternal history of atopic disease, maternal smoking, older sibling, younger sibling, supplement use, and season when the FFQ was completed.

**Supplementary Table E5:** Odds ratio (95% confidence interval) for incident asthma at 11 or 14 years according to quartiles of intakes of preformed vitamin A and β-carotene equivalent, stratified by other *BCMO1* genotypes

| | Quartiles of vitamin A intake | | | | P for | P for |
|---|---|---|---|---|---|---|
| | Q1 | Q2 | Q3 | Q4 | trend* | interaction |
| **Preformed vitamin A** | | | | | | |
| **Upstream *BCMO1*: rs11645428** | | | | | | |
| GG: Cases/non-cases | 40/343 | 23/371 | 35/397 | 33/392 | | |
| aOR (95% CI) | 1.00 | 0.44 (0.25-0.77) | 0.58 (0.34-0.98) | 0.44 (0.24-0.84) | 0.04 | |
| GA: Cases/non-cases | 36/366 | 35/391 | 42/343 | 39/324 | | |
| aOR (95% CI) | 1.00 | 0.96 (0.58-1.59) | 1.31 (0.78-2.19) | 1.23 (0.67-2.26) | 0.37 | 0.23 |
| AA†: Cases/non-cases | 7/ 90 | 8/104 | 5/97 | 10/100 | | |
| aOR (95% CI) | 1.00 | 0.70 (0.18-2.68) | 0.68 (0.15-3.05) | 0.87 (0.19-3.96) | 0.97 | 0.49 |
| **Upstream *BCMO1*: rs6420424** | | | | | | |
| GG†: Cases/non-cases | 26/227 | 22/243 | 21/210 | 21/227 | | |
| aOR (95% CI) | 1.00 | 0.76 (0.40-1.44) | 0.86 (0.44-1.68) | 0.72 (0.33-1.56) | 0.48 | |
| GA: Cases/non-cases | 28/360 | 34/420 | 38/402 | 44/371 | | |
| aOR (95% CI) | 1.00 | 1.09 (0.63-1.88) | 1.18 (0.67-2.05) | 1.36 (0.73-2.55) | 0.31 | 0.09 |
| AA: Cases/non-cases | 29/212 | 10/203 | 23/225 | 17/218 | | |
| aOR (95% CI) | 1.00 | 0.26 (0.12-0.57) | 0.53 (0.27-1.01) | 0.29 (0.13-0.68) | 0.02 | 0.59 |
| ***BCMO1* coding region: rs12934922** | | | | | | |
| AA†: Cases/non-cases | 29/255 | 17/275 | 28/274 | 27/248 | | |
| aOR (95% CI) | 1.00 | 0.62 (0.32-1.19) | 1.09 (0.58-2.03) | 1.33 (0.64-2.74) | 0.26 | |
| AT: Cases/non-cases | 33/383 | 37/431 | 35/414 | 36/380 | | |
| aOR (95% CI) | 1.00 | 0.87 (0.52-1.46) | 0.77 (0.44-1.35) | 0.67 (0.35-1.28) | 0.22 | 0.82 |
| TT: Cases/non-cases | 21/161 | 12/160 | 19/149 | 19/188 | | |
| aOR (95% CI) | 1.00 | 0.49 (0.22-1.09) | 0.77 (0.36-1.62) | 0.48 (0.20-1.14) | 0.18 | 0.63 |
| **β-carotene equivalent** | | | | | | |
| **Upstream *BCMO1*: rs11645428** | | | | | | |
| GG: Cases/non-cases | 35/370 | 27/354 | 40/392 | 29/387 | | |
| aOR (95% CI) | 1.00 | 0.74 (0.44-1.27) | 0.94 (0.57-1.57) | 0.68 (0.39-1.21) | 0.22 | |
| GA: Cases/non-cases | 35/332 | 30/388 | 38/351 | 49/353 | | |
| aOR (95% CI) | 1.00 | 0.80 (0.47-1.34) | 1.09 (0.65-1.84) | 1.38 (0.83-2.28) | 0.10 | 0.09 |
| AA†: Cases/non-cases | 4/ 93 | 6/109 | 7/102 | 13/ 87 | | |
| aOR (95% CI) | 1.00 | 1.51 (0.35-6.46) | 1.85 (0.45-7.64) | 3.77 (0.92-15.4) | 0.04 | 0.01 |
| **Upstream *BCMO1*: rs6420424** | | | | | | |
| GG†: Cases/non-cases | 16/213 | 20/252 | 18/232 | 36/210 | | |
| aOR (95% CI) | 1.00 | 1.25 (0.61-2.56) | 1.31 (0.61-2.79) | 2.84 (1.40-5.77) | 0.001 | |
| GA: Cases/non-cases | 32/376 | 32/377 | 40/393 | 40/407 | | |
| aOR (95% CI) | 1.00 | 0.98 (0.58-1.66) | 1.06 (0.63-1.79) | 1.04 (0.61-1.76) | 0.89 | 0.06 |
| AA: Cases/non-cases | 26/206 | 11/222 | 27/220 | 15/210 | | |
| aOR (95% CI) | 1.00 | 0.35 (0.17-0.75) | 0.85 (0.45-1.59) | 0.49 (0.23-1.03) | 0.12 | 0.004 |
| ***BCMO1* coding region: rs7501331** | | | | | | |
| CC†: Cases/non-cases | 43/483 | 27/500 | 57/504 | 58/469 | | |
| aOR (95% CI) | 1.00 | 0.58 (0.35-0.96) | 1.22 (0.79-1.90) | 1.27 (0.80-2.00) | 0.12 | |
| CT: Cases/non-cases | 27/273 | 29/305 | 25/285 | 24/304 | | |
| aOR (95% CI) | 1.00 | 0.97 (0.54-1.73) | 0.81 (0.43-1.52) | 0.74 (0.39-1.41) | 0.34 | 0.05 |
| TT: Cases/non-cases | 4/ 39 | 7/46 | <5/56 | 9/54 | | |

| | | | | | | |
|---|---|---|---|---|---|---|
| aOR (95% CI) | 1.00 | 5.71 (0.71-46.24) | 2.09 (0.24-18.1) | 12.1 (1.38-106) | 0.03 | 0.99 |

*BCMO1*: β-carotene 15,15'-monooxygenase

[*] Linear trend was tested by treating the median values of quartiles as a continuous variable

[†] Homozygous alleles linked to a more efficient conversion of carotene provitamin A

aOR: Adjusted odds ratio (multivariable model) for sex, total energy intake, maternal education, housing tenure at birth, financial difficulty during pregnancy, maternal ethnicity, maternal history of atopic disease, paternal history of atopic disease, maternal smoking, older sibling, younger sibling, supplement use, and season when the FFQ was completed.

**Supplementary Table E6:** Linear regression coefficients (95% confidence interval) for post-bronchodilator lung function measures (z scores) according to quartiles of intakes of preformed vitamin A, adjusted for further potential confounders

| | Quartiles of vitamin A intake | | | | P for | Per SD |
|---|---|---|---|---|---|---|
| | Q1 | Q2 | Q3 | Q4 | trend* | |
| **Preformed vitamin A** | | | | | | |
| **$FEV_1$** | | | | | | |
| Model 3 | 0.00 | -0.04 (-0.18, 0.10) | -0.03 (-0.18, 0.12) | 0.18 (0.01, 0.35) | 0.03 | 0.08 (0.01, 0.15) |
| Model 4 | 0.00 | -0.02 (-0.16, 0.12) | -0.01 (-0.16, 0.13) | 0.21 (0.04, 0.38) | 0.01 | 0.09 (0.02, 0.15) |
| Model 5 | 0.00 | -0.02 (-0.16, 0.12) | -0.01 (-0.16, 0.13) | 0.21 (0.04, 0.38) | 0.01 | 0.09 (0.02, 0.15) |
| Model 6 | 0.00 | 0.00 (-0.14, 0.14) | -0.01 (-0.15, 0.13) | 0.22 (0.06, 0.39) | 0.007 | 0.09 (0.02, 0.15) |
| Model 7 | 0.00 | -0.01 (-0.16, 0.15) | 0.01 (-0.16, 0.17) | 0.20 (0.02, 0.39) | 0.03 | 0.07 (0.00, 0.15) |
| Model 8 | 0.00 | -0.02 (-0.16, 0.12) | -0.01 (-0.16, 0.14) | 0.22 (0.05, 0.39) | 0.009 | 0.09 (0.02, 0.16) |
| Model 9 | 0.00 | -0.03 (-0.18, 0.11) | -0.04 (-0.19, 0.12) | 0.18 (0.01, 0.35) | 0.03 | 0.08 (0.01, 0.15) |
| Model 10 | 0.00 | -0.03 (-0.17, 0.11) | -0.02 (-0.17, 0.12) | 0.20 (0.04, 0.37) | 0.01 | 0.09 (0.02, 0.15) |
| **FVC** | | | | | | |
| Model 3 | 0.00 | -0.03 (-0.16, 0.11) | -0.05 (-0.19, 0.09) | 0.11 (-0.06, 0.27) | 0.18 | 0.04 (-0.03, 0.10) |
| Model 4 | 0.00 | -0.01 (-0.14, 0.13) | -0.02 (-0.16, 0.11) | 0.15 (-0.02, 0.31) | 0.07 | 0.05 (-0.01, 0.11) |
| Model 5 | 0.00 | -0.01 (-0.14, 0.12) | -0.02 (-0.16, 0.12) | 0.14 (-0.02, 0.30) | 0.07 | 0.05 (-0.01, 0.11) |
| Model 6 | 0.00 | 0.02 (-0.11, 0.15) | -0.02 (-0.16, 0.12) | 0.16 (0.00, 0.32) | 0.05 | 0.05 (-0.01, 0.11) |
| Model 7 | 0.00 | 0.00 (-0.15, 0.15) | -0.00 (-0.16, 0.15) | 0.13 (-0.05, 0.31) | 0.14 | 0.04 (-0.03, 0.11) |
| Model 8 | 0.00 | -0.01 (-0.14, 0.13) | -0.03 (-0.17, 0.11) | 0.15 (-0.02, 0.31) | 0.08 | 0.05 (-0.01, 0.11) |
| Model 9 | 0.00 | -0.03 (-0.17, 0.11) | -0.06 (-0.20, 0.09) | 0.10 (-0.07, 0.26) | 0.21 | 0.04 (-0.02, 0.10) |
| Model 10 | 0.00 | -0.01 (-0.15, 0.12) | -0.03 (-0.17, 0.11) | 0.14 (-0.02, 0.30) | 0.08 | 0.05 (-0.01, 0.11) |
| **$FEV_1$/FVC ratio** | | | | | | |
| Model 3 | 0.00 | 0.03 (-0.09, 0.15) | 0.07 (-0.06, 0.19) | 0.11 (-0.03, 0.26) | 0.11 | 0.06 (0.00, 0.11) |
| Model 4 | 0.00 | 0.01 (-0.11, 0.13) | 0.05 (-0.07, 0.18) | 0.08 (-0.06, 0.23) | 0.20 | 0.05 (-0.01, 0.10) |
| Model 5 | 0.00 | 0.01 (-0.11, 0.13) | 0.05 (-0.07, 0.18) | 0.08 (-0.06, 0.23) | 0.21 | 0.05 (-0.01, 0.10) |
| Model 6 | 0.00 | 0.00 (-0.11, 0.12) | 0.05 (-0.07, 0.18) | 0.08 (-0.06, 0.22) | 0.21 | 0.05 (-0.01, 0.10) |
| Model 7 | 0.00 | 0.01 (-0.12, 0.15) | 0.07 (-0.07, 0.21) | 0.09 (-0.07, 0.25) | 0.21 | 0.03 (-0.03, 0.10) |
| Model 8 | 0.00 | 0.01 (-0.11, 0.13) | 0.06 (-0.06, 0.19) | 0.08 (-0.06, 0.23) | 0.22 | 0.04 (-0.01, 0.10) |
| Model 9 | 0.00 | 0.02 (-0.11, 0.14) | 0.06 (-0.07, 0.19) | 0.10 (-0.05, 0.25) | 0.14 | 0.05 (-0.01, 0.11) |
| Model 10 | 0.00 | 0.01 (-0.11, 0.13) | 0.05 (-0.08, 0.17) | 0.08 (-0.06, 0.22) | 0.23 | 0.05 (-0.01, 0.10) |
| **$FEF_{25–75}$** | | | | | | |
| Model 3 | 0.00 | 0.03 (-0.09, 0.15) | 0.04 (-0.09, 0.16) | 0.17 (0.02, 0.32) | 0.02 | 0.08 (0.03, 0.14) |
| Model 4 | 0.00 | 0.04 (-0.08, 0.16) | 0.04 (-0.08, 0.17) | 0.17 (0.03, 0.31) | 0.02 | 0.08 (0.02, 0.14) |
| Model 5 | 0.00 | 0.04 (-0.08, 0.16) | 0.04 (-0.08, 0.17) | 0.17 (0.03, 0.32) | 0.02 | 0.08 (0.03, 0.14) |
| Model 6 | 0.00 | 0.05 (-0.07, 0.17) | 0.05 (-0.08, 0.17) | 0.18 (0.04, 0.32) | 0.02 | 0.08 (0.03, 0.14) |
| Model 7 | 0.00 | 0.03 (-0.10, 0.17) | 0.09 (-0.05, 0.23) | 0.19 (0.03, 0.35) | 0.02 | 0.07 (0.00, 0.13) |
| Model 8 | 0.00 | 0.03 (-0.09, 0.15) | 0.05 (-0.08, 0.17) | 0.17 (0.03, 0.32) | 0.02 | 0.09 (0.03, 0.14) |
| Model 9 | 0.00 | 0.03 (-0.10, 0.15) | 0.03 (-0.10, 0.16) | 0.16 (0.01, 0.31) | 0.04 | 0.08 (0.02, 0.14) |
| Model 10 | 0.00 | 0.03 (-0.09, 0.15) | 0.04 (-0.09, 0.16) | 0.17 (0.03, 0.31) | 0.02 | 0.08 (0.03, 0.14) |

$FEV_1$: forced expiratory volume in 1s; FVC: forced vital capacity; $FEF_{25–75}$: forced expiratory flow at 25–75% of FVC

* Linear trend was tested by treating the median values of quartiles as a continuous variable
Multivariable model 2 (as presented in table 2): sex and total energy intake, maternal education, housing tenure at birth, financial difficulty during pregnancy, maternal ethnicity, maternal history of atopic disease, paternal history of atopic disease, maternal smoking, older sibling, younger sibling, supplement use, and season when the FFQ was completed.
Multivariable model 3: model 2 plus junk food dietary pattern, traditional dietary pattern, and health-conscious dietary pattern;
Multivariable model 4: model 2 plus any history of food allergy, breastfeeding, and living location (urban vs. rural);
Multivariable model 5: model 2 plus vigorous physical activity;
Multivariable model 6: model 2 plus imputed BMI;
Multivariable model 7: model 2 plus atopy (by skin prick test; n=2,505 for $FEV_1$ and $FEF_{25-75}$ and 2,403 for the rest);
Multivariable model 8: model 2 plus maternal intake of preformed vitamin A and carotene at 32 weeks of gestation;
Multivariable model 9: model 2 plus intakes of vitamins C, D, and E, zinc, protein, and n-3 from fish;
Multivariable model 10: model 2 plus intakes of β-carotene equivalent.

**Supplementary Table E7:** Linear regression coefficients (95% confidence interval) for post-bronchodilator lung function measures (z scores) according to quartiles of intakes of β-carotene equivalent, adjusted for further potential confounders

| | Quartiles of vitamin A intake | | | | P for | Per SD |
|---|---|---|---|---|---|---|
| | Q1 | Q2 | Q3 | Q4 | trend* | |
| **β-carotene equivalent** | | | | | | |
| **$FEV_1$** | | | | | | |
| Model 3 | 0.00 | 0.02 (-0.13, 0.16) | 0.02 (-0.13, 0.18) | -0.08 (-0.25, 0.09) | 0.23 | -0.03 (-0.09, 0.03) |
| Model 4 | 0.00 | 0.07 (-0.07, 0.21) | 0.10 (-0.05, 0.24) | -0.00 (-0.15, 0.15) | 0.70 | -0.00 (-0.06, 0.05) |
| Model 5 | 0.00 | 0.07 (-0.07, 0.21) | 0.10 (-0.04, 0.25) | 0.00 (-0.14, 0.15) | 0.72 | -0.00 (-0.05, 0.05) |
| Model 6 | 0.00 | 0.09 (-0.05, 0.22) | 0.11 (-0.04, 0.25) | 0.02 (-0.12, 0.17) | 0.91 | 0.00 (-0.05, 0.05) |
| Model 7 | 0.00 | 0.03 (-0.12, 0.18) | 0.06 (-0.10, 0.22) | -0.03 (-0.19, 0.14) | 0.56 | -0.00 (-0.06, 0.05) |
| Model 8 | 0.00 | 0.07 (-0.06, 0.21) | 0.09 (-0.05, 0.24) | 0.01 (-0.14, 0.15) | 0.75 | -0.00 (-0.05, 0.05) |
| Model 9 | 0.00 | 0.06 (-0.08, 0.20) | 0.07 (-0.08, 0.22) | -0.03 (-0.19, 0.13) | 0.43 | -0.01 (-0.07, 0.04) |
| Model 10 | 0.00 | 0.08 (-0.06, 0.21) | 0.10 (-0.05, 0.24) | 0.01 (-0.14, 0.16) | 0.79 | -0.00 (-0.05, 0.05) |
| **FVC** | | | | | | |
| Model 3 | 0.00 | -0.02 (-0.16, 0.12) | -0.02 (-0.17, 0.13) | -0.11 (-0.27, 0.05) | 0.14 | -0.04 (-0.10, 0.02) |
| Model 4 | 0.00 | 0.03 (-0.10, 0.16) | 0.06 (-0.08, 0.20) | -0.02 (-0.16, 0.12) | 0.62 | -0.01 (-0.06, 0.04) |
| Model 5 | 0.00 | 0.03 (-0.10, 0.16) | 0.06 (-0.07, 0.20) | -0.01 (-0.16, 0.13) | 0.65 | -0.01 (-0.06, 0.04) |
| Model 6 | 0.00 | 0.05 (-0.08, 0.18) | 0.07 (-0.07, 0.20) | 0.01 (-0.13, 0.15) | 0.84 | -0.00 (-0.05, 0.05) |
| Model 7 | 0.00 | 0.00 (-0.14, 0.15) | 0.05 (-0.11, 0.20) | -0.03 (-0.19, 0.13) | 0.57 | -0.01 (-0.06, 0.05) |
| Model 8 | 0.00 | 0.04 (-0.10, 0.17) | 0.06 (-0.08, 0.19) | -0.01 (-0.15, 0.13) | 0.67 | -0.01 (-0.06, 0.04) |
| Model 9 | 0.00 | 0.01 (-0.12, 0.15) | 0.02 (-0.12, 0.17) | -0.06 (-0.21, 0.09) | 0.32 | -0.02 (-0.07, 0.03) |
| Model 10 | 0.00 | 0.04 (-0.10, 0.17) | 0.06 (-0.08, 0.19) | -0.01 (-0.15, 0.13) | 0.69 | -0.01 (-0.06, 0.04) |
| **$FEV_1$/FVC ratio** | | | | | | |
| Model 3 | 0.00 | 0.07 (-0.05, 0.19) | 0.05 (-0.08, 0.18) | 0.00 (-0.14, 0.15) | 0.71 | 0.00 (-0.05, 0.05) |
| Model 4 | 0.00 | 0.04 (-0.07, 0.16) | 0.01 (-0.11, 0.14) | -0.04 (-0.17, 0.09) | 0.36 | -0.01 (-0.06, 0.03) |
| Model 5 | 0.00 | 0.05 (-0.07, 0.17) | 0.02 (-0.10, 0.14) | -0.04 (-0.17, 0.08) | 0.32 | -0.01 (-0.06, 0.03) |
| Model 6 | 0.00 | 0.04 (-0.07, 0.16) | 0.02 (-0.10, 0.14) | -0.05 (-0.17, 0.08) | 0.28 | -0.02 (-0.06, 0.03) |
| Model 7 | 0.00 | 0.04 (-0.09, 0.17) | -0.01 (-0.14, 0.13) | -0.08 (-0.22, 0.07) | 0.17 | -0.02 (-0.07, 0.03) |
| Model 8 | 0.00 | 0.05 (-0.07, 0.17) | 0.01 (-0.11, 0.14) | -0.04 (-0.17, 0.08) | 0.33 | -0.01 (-0.06, 0.03) |
| Model 9 | 0.00 | 0.06 (-0.06, 0.18) | 0.04 (-0.09, 0.17) | -0.02 (-0.15, 0.12) | 0.53 | -0.01 (-0.05, 0.04) |
| Model 10 | 0.00 | 0.05 (-0.07, 0.17) | 0.02 (-0.11, 0.14) | -0.04 (-0.17, 0.09) | 0.33 | -0.01 (-0.06, 0.03) |
| **$FEF_{25–75}$** | | | | | | |
| Model 3 | 0.00 | 0.06 (-0.06, 0.19) | 0.06 (-0.07, 0.20) | 0.01 (-0.13, 0.16) | 0.82 | -0.00 (-0.05, 0.05) |
| Model 4 | 0.00 | 0.07 (-0.05, 0.19) | 0.07 (-0.05, 0.20) | 0.01 (-0.11, 0.14) | 0.85 | -0.00 (-0.04, 0.04) |
| Model 5 | 0.00 | 0.08 (-0.04, 0.20) | 0.08 (-0.04, 0.21) | 0.02 (-0.11, 0.15) | 0.88 | 0.00 (-0.04, 0.05) |
| Model 6 | 0.00 | 0.08 (-0.04, 0.20) | 0.08 (-0.04, 0.21) | 0.03 (-0.10, 0.15) | 0.97 | 0.00 (-0.04, 0.05) |
| Model 7 | 0.00 | 0.06 (-0.08, 0.19) | 0.05 (-0.09, 0.18) | -0.01 (-0.15, 0.14) | 0.68 | -0.00 (-0.05, 0.05) |
| Model 8 | 0.00 | 0.07 (-0.04, 0.19) | 0.07 (-0.05, 0.19) | 0.01 (-0.11, 0.14) | 0.87 | 0.00 (-0.04, 0.05) |
| Model 9 | 0.00 | 0.07 (-0.05, 0.19) | 0.07 (-0.05, 0.20) | 0.01 (-0.13, 0.14) | 0.73 | -0.00 (-0.05, 0.04) |
| Model 10 | 0.00 | 0.08 (-0.04, 0.20) | 0.07 (-0.05, 0.20) | 0.02 (-0.11, 0.15) | 0.92 | 0.00 (-0.04, 0.05) |

$FEV_1$: forced expiratory volume in 1s; FVC: forced vital capacity; $FEF_{25–75}$: forced expiratory flow at 25–75% of FVC

* Linear trend was tested by treating the median values of quartiles as a continuous variable
Multivariable model 2 (as presented in table 2): sex and total energy intake, maternal education, housing tenure at birth, financial difficulty during pregnancy, maternal ethnicity, maternal history of atopic disease, paternal history of atopic disease, maternal smoking, older sibling, younger sibling, supplement use, and season when the FFQ was completed.
Multivariable model 3: model 2 plus junk food dietary pattern, traditional dietary pattern, and health-conscious dietary pattern;
Multivariable model 4: model 2 plus any history of food allergy, breastfeeding, and living location (urban vs. rural);
Multivariable model 5: model 2 plus vigorous physical activity;
Multivariable model 6: model 2 plus imputed BMI;
Multivariable model 7: model 2 plus atopy (by skin prick test; n=2,505 for $FEV_1$ and $FEF_{25-75}$ and 2,403 for the rest);
Multivariable model 8: model 2 plus maternal intake of preformed vitamin A and carotene at 32 weeks of gestation;
Multivariable model 9: model 2 plus intakes of vitamins C, D, and E, zinc, protein, and n-3 from fish;
Multivariable model 10: model 2 plus intakes of β-carotene equivalent.

**Supplementary Table E8:** Odds ratio (95% confidence interval) for incident asthma at 11 or 14 years according to quartiles of intakes of preformed vitamin A and β-carotene equivalent, adjusted for further potential confounders

| | Quartiles of vitamin A intake | | | | P for trend* | Per SD |
|---|---|---|---|---|---|---|
| | **Q1** | **Q2** | **Q3** | **Q4** | | |
| **Preformed vitamin A** | | | | | | |
| Model 3 | 1.00 | 0.74 (0.55-1.01) | 0.78 (0.57-1.07) | 0.65 (0.44-0.94) | 0.04 | 0.80 (0.68, 0.94) |
| Model 4 | 1.00 | 0.77 (0.57-1.04) | 0.81 (0.59-1.11) | 0.68 (0.47-0.99) | 0.07 | 0.81 (0.69, 0.96) |
| Model 5 | 1.00 | 0.77 (0.57-1.05) | 0.81 (0.59-1.11) | 0.68 (0.47-0.99) | 0.07 | 0.82 (0.70, 0.96) |
| Model 6 | 1.00 | 0.77 (0.57-1.04) | 0.81 (0.59-1.10) | 0.68 (0.47-0.99) | 0.07 | 0.82 (0.70, 0.96) |
| Model 7 | 1.00 | 0.66 (0.46-0.96) | 0.71 (0.49-1.04) | 0.66 (0.42-1.02) | 0.12 | 0.82 (0.67, 0.99) |
| Model 8 | 1.00 | 0.74 (0.54-1.02) | 0.80 (0.58-1.10) | 0.68 (0.46-0.99) | 0.07 | 0.83 (0.70, 0.98) |
| Model 9 | 1.00 | 0.73 (0.54-1.01) | 0.79 (0.57-1.09) | 0.69 (0.47-1.01) | 0.10 | 0.83 (0.70, 0.97) |
| Model 10 | 1.00 | 0.78 (0.58-1.06) | 0.81 (0.59-1.11) | 0.68 (0.47-0.98) | 0.06 | 0.82 (0.70, 0.96) |
| **β-carotene equivalent** | | | | | | |
| Model 3 | 1.00 | 0.74 (0.53-1.03) | 1.05 (0.75-1.47) | 1.08 (0.75-1.56) | 0.38 | 1.06 (0.92, 1.21) |
| Model 4 | 1.00 | 0.80 (0.58-1.10) | 1.14 (0.84-1.54) | 1.15 (0.83-1.58) | 0.24 | 1.06 (0.95, 1.19) |
| Model 5 | 1.00 | 0.79 (0.57-1.08) | 1.13 (0.83-1.54) | 1.16 (0.84-1.59) | 0.20 | 1.07 (0.96, 1.20) |
| Model 6 | 1.00 | 0.80 (0.58-1.09) | 1.14 (0.84-1.55) | 1.16 (0.84-1.59) | 0.20 | 1.07 (0.96, 1.20) |
| Model 7 | 1.00 | 0.80 (0.54-1.18) | 1.24 (0.86-1.79) | 1.21 (0.82-1.77) | 0.21 | 1.09 (0.95, 1.25) |
| Model 8 | 1.00 | 0.82 (0.59-1.13) | 1.17 (0.86-1.59) | 1.16 (0.84-1.59) | 0.23 | 1.06 (0.95, 1.19) |
| Model 9 | 1.00 | 0.77 (0.55-1.07) | 1.14 (0.83-1.57) | 1.14 (0.82-1.60) | 0.25 | 1.07 (0.95, 1.20) |
| Model 10 | 1.00 | 0.82 (0.59-1.12) | 1.17 (0.86-1.60) | 1.18 (0.85-1.62) | 0.19 | 1.07 (0.96, 1.20) |

* Linear trend was tested by treating the median values of quartiles as a continuous variable
Multivariable model 2 (as presented in table 2): sex and total energy intake, maternal education, housing tenure at birth, financial difficulty during pregnancy, maternal ethnicity, maternal history of atopic disease, paternal history of atopic disease, maternal smoking, older sibling, younger sibling, supplement use, and season when the FFQ was completed.
Multivariable model 3: model 2 plus junk food dietary pattern, traditional dietary pattern, and health-conscious dietary pattern;
Multivariable model 4: model 2 plus any history of food allergy, breastfeeding, and living location (urban vs. rural);
Multivariable model 5: model 2 plus vigorous physical activity;
Multivariable model 6: model 2 plus imputed BMI;
Multivariable model 7: model 2 plus atopy (by skin prick test; n=3,346);
Multivariable model 8: model 2 plus maternal intake of preformed vitamin A and carotene at 32 weeks of gestation;
Multivariable model 9: model 2 plus intakes of vitamins C, D, and E, zinc, protein, and n-3 from fish;
Multivariable model 10: model 2 plus intakes of β-carotene equivalent.

**Supplementary Table E9:** Associations of energy adjusted intakes of preformed vitamin A and β-carotene equivalent using residual method with incident asthma at 11 or 14 years and post-bronchodilator lung function measures (z scores), adjusted for potential confounders

| | Quintiles of vitamin A intake | | | | | | |
|---|---|---|---|---|---|---|---|
| | **Q1** | **Q2** | **Q3** | **Q4** | **Q5** | **P-trend** | **Per SD** |
| **Preformed vitamin A** | | | | | | | |
| Median (IQR), µg/d | 304 (263-330) | 383 (368-398) | 438 (425-451) | 498 (482-516) | 601 (565-670) | | |
| **Incident asthma** | | | | | | | |
| Cases/non-cases | 84/732 | 72/828 | 84/871 | 81/864 | 69/855 | | |
| aOR (95% CI) | 1.00 | 0.80 (0.57-1.11) | 0.84 (0.61-1.17) | 0.83 (0.60-1.16) | 0.70 (0.50-0.98) | 0.06 | 0.85 (0.75, 0.97) |
| **$FEV_1$** | | | | | | | |
| aβ (95% CI) | 0.00 | -0.02 (-0.18, 0.13) | 0.01 (-0.14, 0.17) | 0.00 (-0.15, 0.16) | 0.13 (-0.02, 0.29) | 0.06 | 0.07 (0.02, 0.12) |
| **FVC** | | | | | | | |
| aβ (95% CI) | 0.00 | -0.00 (-0.15, 0.14) | 0.05 (-0.10, 0.20) | -0.02 (-0.17, 0.13) | 0.09 (-0.06, 0.23) | 0.30 | 0.04 (-0.01, 0.09) |
| **$FEV_1$/FVC ratio** | | | | | | | |
| aβ (95% CI) | 0.00 | -0.02 (-0.15, 0.11) | -0.07 (-0.20, 0.07) | 0.02 (-0.11, 0.15) | 0.07 (-0.06, 0.20) | 0.18 | 0.04 (-0.01, 0.08) |
| **$FEF_{25-75}$** | | | | | | | |
| aβ (95% CI) | 0.00 | -0.01 (-0.14, 0.12) | -0.03 (-0.16, 0.11) | -0.00 (-0.14, 0.13) | 0.12 (-0.01, 0.25) | 0.05 | 0.07 (0.02, 0.11) |
| **β-carotene equivalent** | | | | | | | |
| Median (IQR), µg/d | 958 (710-1181) | 1570 (1487-1639) | 1811 (1756-1870) | 2088 (1997-2206) | 3353 (3033-3608) | | |
| **Incident asthma** | | | | | | | |
| Cases/non-cases | 76/798 | 62/798 | 72/835 | 87/866 | 93/853 | | |
| aOR (95% CI) | 1.00 | 0.80 (0.56-1.14) | 0.97 (0.69-1.36) | 1.09 (0.79-1.52) | 1.17 (0.84-1.61) | 0.13 | 1.06 (0.96, 1.18) |
| **$FEV_1$** | | | | | | | |
| aβ (95% CI) | 0.00 | 0.09 (-0.07, 0.25) | 0.01 (-0.15, 0.16) | 0.10 (-0.05, 0.26) | -0.03 (-0.18, 0.13) | 0.49 | -0.00 (-0.05, 0.05) |
| **FVC** | | | | | | | |
| aβ (95% CI) | 0.00 | 0.05 (-0.10, 0.20) | -0.02 (-0.17, 0.13) | 0.13 (-0.02, 0.27) | -0.07 (-0.21, 0.08) | 0.29 | -0.00 (-0.05, 0.04) |
| **$FEV_1$/FVC ratio** | | | | | | | |
| aβ (95% CI) | 0.00 | 0.06 (-0.07, 0.20) | -0.02 (-0.16, 0.11) | -0.11 (-0.25, 0.02) | -0.01 (-0.14, 0.12) | 0.57 | -0.01 (-0.06, 0.03) |
| **$FEF_{25-75}$** | | | | | | | |
| aβ (95% CI) | 0.00 | 0.13 (-0.01, 0.26) | -0.02 (-0.15, 0.11) | -0.00 (-0.13, 0.13) | 0.02 (-0.11, 0.15) | 0.83 | 0.00 (-0.04, 0.04) |

[*] Linear trend was tested by treating the median values of quartiles as a continuous variable

aOR and aβ: Adjusted odds ratio and linear regression coefficients (in multivariable model) for sex, total energy intake, maternal education, housing tenure at birth, financial difficulty during pregnancy, maternal ethnicity, maternal history of atopic disease, paternal history of atopic disease, maternal smoking, older sibling, younger sibling, supplement use, and season when the FFQ was completed.
