# Supplementary material for: Dietary intake of vitamin A, lung function and incident asthma in childhood
Source: Eur Respir J. 2021 Oct 28;58(4):2004407. doi: 10.1183/13993003.04407-2020 (PMC8551559; doi:10.1183/13993003.04407-2020)
Supplement: Supplementary file 2 [file ERJ-04407-2020.Shareable.pdf]

EUROPEAN RESPIRATORY JOURNAL
ORIGINAL RESEARCH ARTICLE
M. TALAEI ET AL.

SHAREABLE PDF

Dietary intake of vitamin A, lung function and incident asthma in childhood

Mohammad Talaei [1], David A. Hughes [2], Osama Mahmoud [2], Pauline M. Emmett [3], Raquel Granell [2], Stefano Guerra [4] and Seif O. Shaheen [1]

[1]Institute of Population Health Sciences, Barts and The London School of Medicine and Dentistry, Queen Mary University of London, London, UK. [2]MRC Integrative Epidemiology Unit, Population Health Sciences, Bristol Medical School, University of Bristol, Bristol, UK. [3]Centre for Academic Child Health, Population Health Sciences, Bristol Medical School, University of Bristol, Bristol, UK. [4]Asthma and Airway Disease Research Center, University of Arizona, Tucson, AZ, USA.

Corresponding author: Mohammad Talaei (m.talaei@qmul.ac.uk)

Check for updates

Shareable abstract (@ERSpublications)
A higher intake of preformed vitamin A, but not pro-vitamin β-carotene, in mid-childhood was associated with higher subsequent lung function and lower risk of fixed airflow limitation and incident asthma https://bit.ly/3d7PUca

Cite this article as: Talaei M, Hughes DA, Mahmoud O, *et al*. Dietary intake of vitamin A, lung function and incident asthma in childhood. *Eur Respir J* 2021; 58: 2004407 [DOI: 10.1183/13993003.04407-2020].

This single-page version can be shared freely online.

Copyright ©The authors 2021.

This version is distributed under the terms of the Creative Commons Attribution Licence 4.0.

This article has supplementary material available from erj.ersjournals.com

Received: 03 Dec 2020
Accepted: 18 March 2021

Abstract

Background Longitudinal epidemiological data are scarce on the relationship between dietary intake of vitamin A and respiratory outcomes in childhood. We investigated whether a higher intake of preformed vitamin A or pro-vitamin β-carotene in mid-childhood is associated with higher lung function and with asthma risk in adolescence.

Methods In the Avon Longitudinal Study of Parents and Children, dietary intakes of preformed vitamin A and β-carotene equivalents were estimated by food frequency questionnaire at 7 years of age. Post-bronchodilator forced expiratory volume in 1 s (FEV_1), forced vital capacity (FVC) and forced expiratory flow at 25–75% of FVC ($FEF_{25-75\%}$) were measured at 15.5 years and transformed to z-scores. Incident asthma was defined by new cases of doctor-diagnosed asthma at age 11 or 14 years.

Results In multivariable adjusted models, a higher intake of preformed vitamin A was associated with higher lung function and a lower risk of incident asthma: comparing top *versus* bottom quartiles of intake, regression coefficients for FEV_1 and $FEF_{25-75\%}$ were 0.21 (95% CI 0.05–0.38; p_{trend}=0.008) and 0.18 (95% CI 0.03–0.32; p_{trend}=0.02), respectively; odds ratios for FEV_1/FVC below the lower limit of normal and incident asthma were 0.49 (95% CI 0.27–0.90; p_{trend}=0.04) and 0.68 (95% CI 0.47–0.99; p_{trend}=0.07), respectively. In contrast, there was no evidence for association with β-carotene. We also found some evidence for modification of the associations between preformed vitamin A intake and lung function by *BCMO1*, *NCOR2* and *SCGB1A1* gene polymorphisms.

Conclusion A higher intake of preformed vitamin A, but not β-carotene, in mid-childhood is associated with higher subsequent lung function and lower risk of fixed airflow limitation and incident asthma.
